# Analyzing lognormal data: A nonmathematical practical guide

**DOI:** 10.1016/j.pharmr.2025.100049

**Published:** 2025-02-25

**Authors:** Harvey J. Motulsky, Trajen Head, Paul B.S. Clarke

**Affiliations:** 1 GraphPad Software, Los Angeles, California; 2 GraphPad Software, Boston, Massachusetts; 3 Department of Pharmacology and Therapeutics, McGill University, Montreal, Quebec, Canada

## Abstract

Lognormal distributions are pervasive in pharmacology and elsewhere in biomedical science, arising naturally when biological effects multiply rather than add. Despite their ubiquity in pharmacological parameters (eg, EC50, IC50, Kd, and Km), lognormal distributions are often overlooked or misunderstood, leading to flawed data analysis. This largely nonmathematical review explains why lognormal distributions are common, how to recognize them, and how to analyze them appropriately. We show that many measured variables are lognormal. So are many derived parameters, particularly those defined as ratios of lognormal variables. Through examples and simulations accessible to working scientists, we demonstrate how misidentifying lognormal distributions as normal leads to reduced statistical power, unnecessarily large sample sizes, false identification of outliers, and inappropriate reporting of effects as differences rather than ratios. We challenge the common practice of using normality tests to decide how to analyze data, showing that many data sets pass both normality and lognormality tests, especially with small sample sizes. Instead, we advocate for assuming lognormality based on the nature of the variable. This review provides practical guidance on recognizing and presenting lognormal data, and comparing data sets sampled from lognormal distributions. Based on Monte Carlo simulations, we recommend the lognormal Welch’s *t* test or nonparametric Brunner-Munzel test for comparing 2 unpaired groups, the lognormal ratio paired *t* test for paired comparisons, and lognormal ANOVA for ≥3 groups. By recognizing and properly handling lognormal distributions, pharmacologists can design more efficient experiments, obtain more reliable statistical inferences, and communicate their results more effectively.

**Significance Statement:**

Lognormal distributions are common in pharmacology and many scientific fields, but they are often misunderstood or overlooked. This review provides a detailed guide to recognizing and analyzing lognormal data, aiming to help pharmacologists perform more appropriate and more powerful statistical analyses, draw more meaningful conclusions from their data, and communicate their results more effectively.

## I Introduction

### A motivating example

A

**Fig. 1:**
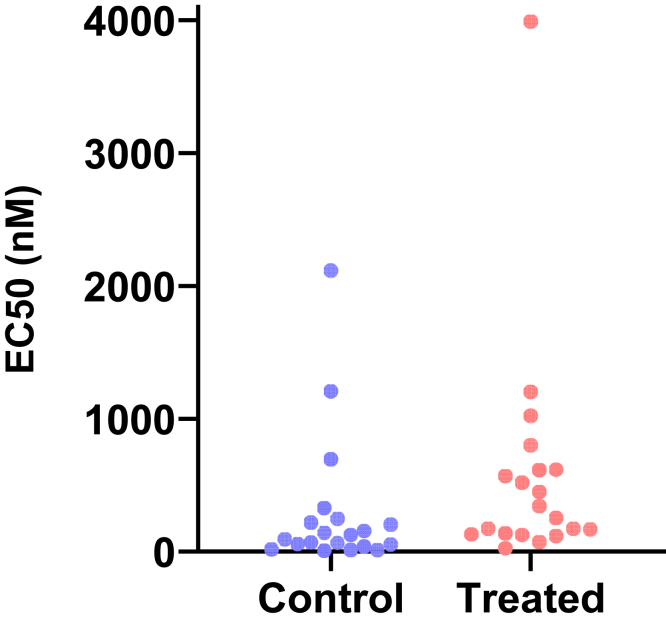
Sample data to demonstrate why it is important to identify lognormal distributions. These values were obtained by first randomly choosing values from lognormal distributions with GeoSD = 4 and GeoMeans = 100 and 400, and then rounding each value to the nearest integer (to make it easy for readers to analyze with their own software). The raw data are in Supplemental Material.

This motivating example demonstrates that analyzing lognormal data as if the values were sampled from a normal distribution can lead to incorrect and misleading conclusions. Figure 1 compares EC50 values for control and treated conditions.

#### 1 Incorrect analysis assuming sampling from normal distributions

Here are the results if the data were analyzed conventionally with a 2-sample unpaired *t* test assuming sampling from normal distributions:

• The means are 294 nM (control) and 575 nM (treated). The difference is 282 nM (95% confidence interval [CI] of the difference: −175 to 739 nM). With such a wide CI), the data are consistent with no difference, a moderate decrease, or a large increase. In other words, no conclusion is possible. Note, here we refer to the arithmetic mean (AMean)—in other words, the type of mean that everyone is familiar with. We will get to the geometric mean in the next section.
• The *P* value (two-tailed) testing the null hypothesis that the sets of data were sampled from identical normal distributions is 0.22. This is greater than the traditional threshold of 0.05, so the null hypothesis of no difference would not be rejected.
• Looking at the graph, the largest value in each group is much larger than the rest. Indeed, Grubbs’ (1969) outlier test with *α* set to 0.05 identified an outlier in each case.

#### 2 Correct analysis assuming sampling from lognormal distributions

Now let us analyze correctly, assuming sampling from lognormal distributions, using methods that will be explained in detail below.

• First, a quick reminder about samples and distributions. Commonly used statistical tests (such as *t* tests) proceed by assuming the null hypothesis, that is, that there is no real difference between conditions (here, control vs treated). These tests then ask how frequently such an extreme (or even more extreme) result would be obtained by taking random samples from an invisible population (distribution) comprising all analogous experiments that you could have performed if the null hypothesis were true.
• Geometric means (GeoMeans), explained below, are estimates of the medians of the distributions that the data were sampled from. The GeoMeans are 103 nM (control) and 302 nM (treated). The ratio is 2.9 with a 95% CI of the ratio ranging from 1.24 to 6.93.
• The *P* value (two-tailed) testing the null hypothesis that both populations are identical and lognormal is 0.015. This is less than the traditional threshold of 0.05 so the null hypothesis would be rejected.
• The section The lognormal Welch’s t test will explain an alternative (or extension).
• After log-transforming the raw data, Grubbs’ outlier test (*α* = 0.05) did not identify an outlier in either group.

#### 3 Why it can matter

For this example, analyzing the data correctly solved 3 problems:

• Reporting the experimental effect as a ratio is scientifically sensible. The treatment approximately triples the EC50, and the CI tells us the data are compatible with a ratio between a 1.2 and 6.9.
• With the correct analysis, the two-tailed *P* value is .015. Because this is less than the .05 threshold many scientists routinely use, the null hypothesis of no treatment effect would be rejected. Assuming lognormal distributions reversed the conclusion of this experiment.
• Grubbs’ test detected no outliers when done properly on log-transformed values.

We will return to this example in the section Why the unpaired t test (without log transformation) should be avoided with lognormal data.

### B History of lognormal distributions

Lognormal distributions, first described in 1879 (Galton, 1879; McAlister, 1879) are asymmetrical distributions commonly encountered in many fields of science. Despite their long history and widespread occurrence, they are often overlooked or misunderstood. Aitchison and Brown (1957) designated the lognormal distribution as the “Cinderella of distributions,” shunned compared with its normal “sister,” and many scientists still mistakenly consider lognormality to be an obscure topic that can generally be ignored. Many biostatistics texts do not even mention lognormal distributions or geometric means (Zar, 2009; Glantz, 2011; Dancey et al, 2012; Irizarry and Love, 2016; Baldi and Moore, 2017; Daniels and Cross, 2018; Glaser, 2018). We only know of a few biostatistics texts that devote more than a few pages to this topic (Bland, 2015; Motulsky, 2017; Wahi and Puzzullo, 2024).

### C Our goals in writing this review

Prior reviews have urged scientists to realize how common lognormal distributions are, and how much is gained by analyzing lognormal data properly. These reviews, however, do not provide a useful guide for working scientists to analyze lognormal data because they are either too concise (Gaddum, 1945; Limpert et al, 2001; Limpert and Stahel, 2011, 2017; Curran-Everett, 2018) or too mathematical (Crow and Shimizu, 1988; Parkin and Robinson, 1992; Vogel, 2022; https://en.wikipedia.org/wiki/Log-normal_distribution).

In this review we aim to clarify the concept of lognormal distributions, explain their relevance in pharmacology, and provide guidance on how to work with these distributions. We limit our discussion to describing lognormal distributions and comparing data sets sampled from such distributions. We do not discuss the use of lognormal distributions in regression or in Bayesian analyses. Neither do we discuss 3-parameter (ie, “shifted”) lognormal distributions, which can occur when there is a defined minimum value other than zero; although such variables are encountered in medicine (Royston, 1992) they are rarely relevant to pharmacology.

We point out that failing to recognize a lognormal distribution can lead to erroneous conclusions and misinterpretation of data. We also show that normality and lognormality tests are nowhere near as useful as many expect, and so we recommend that lognormal distributions be assumed (without testing) for many of the variables measured by pharmacologists. Some readers may prefer to skip ahead to our list of recommendations in the Summary.

## II Ratio scale variables

All lognormal variables are ratio scale variables, so let us start there.

### A Definition of ratio scale variables

Many variables measured and compared in pharmacology are ratio scale variables with the following properties (Stevens, 1946):

• Negative values are inconceivable. Only positive values are possible.
• Zero either means none of that variable or the asymptotic value the variable can approach. Examples of the former are weight or length. A weight of zero means no weight. A height of zero means no height. Examples of the latter are an EC50 or K_m_ value. It is not possible for either of those parameters to equal zero, but their values can be tiny and approach zero.
• Converting between units requires only multiplication or division, as is the case for weights, concentrations, durations, lengths, and EC50s.
• It makes scientific sense to calculate a ratio. For example, 4 cm is twice as long as a distance of 2 cm; 6 L of water is 3 times as much water as 2 L; and an EC50 of 5 *μ*M is 5 times greater than an EC50 of 1 *μ*M.

### B Examples of ratio variables and a counterexample

• Enzyme velocities can only be positive, with zero indicating no enzyme activity. If an enzyme velocity is reduced from 100 to 50 units (U)/min, it is best quantified as a ratio. The velocity was cut in half. If the same inhibitor in a different system reduces velocity from 50 to 25 U/min, the effect would be seen as the same: the inhibitor cuts the enzyme velocity in half. The fact that the differences are distinct (a change of 50 U/min vs a change of 25 U/min) would not usually be relevant.
• Equilibrium dissociation constants (Kd) can only have positive values. They cannot equal zero but can have any positive value. If changing one amino acid in a receptor molecule alters the Kd of agonist binding from 5 to 1 *μ*M, the finding is best summarized as a ratio—the Kd was reduced by a factor of 5 or (equivalently) the affinity went up by a factor of 5. The difference (4 *μ*M) is not interesting and would not be reported.
• Temperature in either Celsius or Fahrenheit is a counterexample as it does not meet any of the 4 criteria defining a ratio variable. Negative values are meaningful. A temperature of 0 °C or 0 °F does not mean “no temperature.” Converting between the 2 temperature scales requires more than just multiplication or division. Ratios are not meaningful (200 °C is not twice as hot as 100 °C) so changes in temperature in °C or °F must be expressed as differences. For all 4 reasons, temperature in °C or °F is not a ratio variable. In contrast, temperature in kelvin is a ratio variable because it has a true zero (absolute zero) and ratios are meaningful.

### C For ratio variables, experimental effects are best reported as ratios, not differences

When comparing the effects of different factors on a ratio variable, it is usually more meaningful to focus on (or report) the relative change (ratio) rather than the absolute change (difference).

Consider the following example: a drug increases your measure from 5 to 10 U in first subject, from 10 to 20 U in a second subject, and from 7 to 14 U in a third individual. These 3 results would be seen as identical—the drug has a doubling effect. In other words, the underlying mechanism affecting changes in the variable is *multiplicative*. The relative change is the same, regardless of the starting value. The unequal absolute differences (an increase of 5 U vs 10 U vs 7 U) are most likely irrelevant.

## III Lognormal distributions

Variables on a ratio scale can follow various distributions, including a Poisson distribution for counted variables, and an exponential or γ distribution for waiting times. However, many ratio scale variables are distributed as a lognormal distribution, as elaborated below.

### A Multiplicative causes of variation lead to asymmetrical distributions

**Fig. 2:**
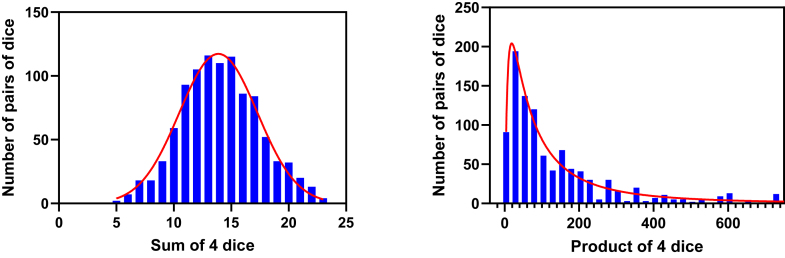
Multiplicative factors lead to an asymmetrical distribution. The graphs show simulations of throwing 4 dice 1000 times. The graph on the left is a frequency distribution of the sum of the values appearing on 4 dice. The graph on the right is a frequency distribution of the product of the 4 values. Our simulations were inspired by a blog by M.H. Nederlof (https://www.mhnederlof.nl/lognormal.html).

Lognormal distributions can arise when the effects of different factors on the variable are multiplicative. Figure 2 simulates throwing 4 dice, computing their sum and product, repeating 1000 times, and tabulating the distribution of *sums* (left graph; symmetrical) and *products* (right graph; asymmetrical). This demonstrates that multiplicative variation leads to an asymmetrical distribution.

To understand why multiplicative factors lead to skewed distributions, consider an example where a variable with an average value of 10 is randomly doubled or halved. Doubling the value results in 20 (an increase of 10), whereas halving it results in 5 (a decrease of 5). The magnitude of the increase is greater than that of the decrease, despite the symmetry of the multiplicative factor. This asymmetry in the magnitudes of change leads to a skewed distribution when compounded over many instances.

Another perspective is to consider the ratios between the values. A ratio of 1.0 represents no change from the average. Doubling the average yields a ratio of 2.0, whereas halving it results in a ratio of 0.5. These ratios are not equidistant from 1.0 on a linear scale—2.0 is farther from 1.0 than is 0.5. This asymmetry in the ratios explains the skewed distribution caused by multiplicative errors.

Lognormal distributions can arise for reasons other than multiplicative error. Koch (1966) reviews several mechanisms that lead to lognormal distributions, and separately (Koch, 1969) reviews mechanisms that create distributions that are not lognormal but look very similar.

### B Review of logarithms

Logarithms have the unique ability to transform multiplicative relationships into additive ones, because the logarithm of a product equals the sum of the logarithms of its factors:

log(a×b)=log(a)+log(b).

The logarithm (base 10) of 1000 is the power of 10 that equals 1000. The logarithm of 1000 is 3, because 10^3^ = 1000. The logarithm of 10 is 1, because 10^1^ = 10. The logarithm of 0.001 is −3, because 10^−3^ = 1/10^3^ = 0.001. The logarithm of 3.162 is 0.5, because 10^0.5^ = $\sqrt{10}$ = 3.162. The logarithm of 1.0 is 0.0, because 10^0^ = 1.0.

The above logarithms are base 10 logarithms, also called *common logarithms*, because the computations take 10 to some power. They are sometimes written as “log10(x).” Mathematicians prefer natural logarithms using base e (2.718. . .), written as ln(x). Beware of the notation “log(x),” which can mean either common or natural logarithm, depending on the field or program.

The logarithms of values > 1.0 are positive. The logarithms of values >0.0 and <1.0 are negative. The logarithms of zero and all negative numbers are simply undefined, because there is no power of 10 that results in a negative number or zero.

### C Logarithms convert a skewed distribution caused by multiplicative error to a symmetrical distribution

Revisiting the earlier example where a factor randomly doubles or halves a value of 10, resulting in 20 or 5, the logarithms (base 10) of these values and the differences between them are:

log(20)≈1.30

log(10)=1

log(5)≈0.70

log(20)−log(10)≈0.30

$$\log\left(10\right)-\log\left(5\right)\approx 0.30$$

The 2 differences on the logarithmic scale are equidistant from log(1.0) = 0.0, demonstrating that the logarithmic transformation eliminates the asymmetry on the original scale, making the effect of the multiplicative factor symmetrical on the logarithmic scale.

### D Relationships between normal and lognormal distributions

The relationships between normal and lognormal distributions are simple.

• If an infinite population of values define a lognormal distribution, then the logarithms of those values define a normal distribution.
• If an infinite population of values define a normal distribution, then the antilogarithms of those values define a lognormal distribution. The antilogarithm is the inverse of the logarithm. The antilogarithm of a common logarithm equals 10 to that power. For example, the antilogarithm of 3 is 10^3^ or 1000. The antilogarithm of a natural logarithm equals e to that power. For example, the antilogarithm of 6.908 is e^6.908^, often written as exp(6.908), which is 1000.

The term “lognormal,” often written “log-normal,” is potentially confusing as it can be incorrectly thought of as the “log of normal.” But it is a mistake to think that values in a lognormal distribution are the logarithms of values from a normal distribution. We show it struck out because it is simply wrong. The term “antilognormal” describes the distribution better than “lognormal” (Johnson et al, 1994), but that term is rarely (if ever) used.

**Fig. 3:**
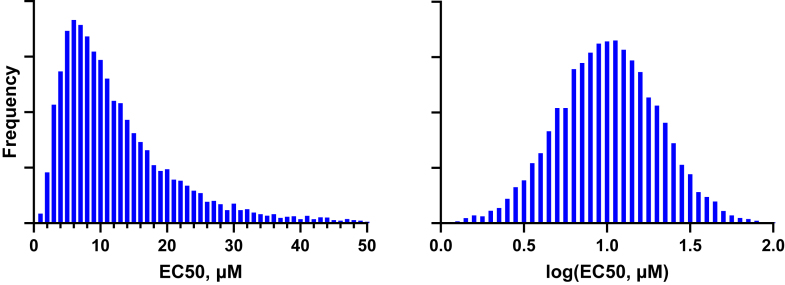
Frequency distribution of a lognormal distribution. Left: A frequency distribution of a lognormal distribution with GeoMean = 10 and GeoSD = 2, defined later in this article. Right: Frequency distribution of the common (base 10) logarithms of the values. Note that the log transformation converts the asymmetrical lognormal distribution to a symmetrical normal distribution.

The distribution of lognormal values is asymmetrical with a positive skew, that is, with a long tail to the right (but as we will see, this asymmetry can be subtle in some cases). However, the distribution of their logarithms is symmetrical and forms a normal (ie, Gaussian) distribution, as shown in Fig. 3.

### E Lognormal distributions are common in biology and beyond

There seems to be a prevailing sense that experimental data usually follow a normal distribution. But it has been realized for a century that this is not true. A 75-year-old text states, “The normal curve was, in fact, to the early statisticians what the circle was to the Ptolemaic astronomers” (Yule and Kendall, 1950). We now know that planets move in ellipses rather than circles, and that lognormal distributions are pervasive.

Lognormal distributions have been noted in data sets ranging from journal citation counts (Thelwall, 2016) to professorial salaries (Benzidia and Lubrano, 2020), and from pollution levels in Los Angeles to the number of words in telephone conversations (Limpert et al, 2001). Examples from biology and medicine include: neuronal firing rates in the central nervous system (Buzsáki and Mizuseki, 2014); numerous blood analytes including triglycerides (Carlson, 1960), free fatty acids (Heath, 1967), ferritin (Custer et al, 1995), alkaline phosphatase, creatinine, glucose, and iron (Flynn et al, 1974); abdominal fat (Stanforth et al, 2004), senile plaque size in Alzheimer disease (Hyman et al, 1995), blood pressure (Bodey and Michell, 1996), the number of parasites per host (Shaw and Dobson, 1995), and cancer survival times (Qazi et al, 2007). For yet more examples, see Limpert et al (2001).

### F EC50, IC50, Kd, K_m_ (and more) tend to be lognormal

#### 1 Data demonstrating pharmacological parameters are lognormal

Gaddum (1945) was probably the first to emphasize the importance of lognormal distributions in pharmacology. Since then, several pharmacological parameters have been shown to be distributed in a lognormal fashion. Table 1 lists pharmacodynamic measures (notably EC50, IC50, Kd, K_i_, and Hill coefficient). Table 2 lists pharmacokinetic measures such as area under the curve, clearance, volume of distribution, elimination half-life, plasma, and intracellular concentrations.

**Table 1:** Pharmacodynamic parameters reported to follow lognormal distributions

Parameter	Parameter/Topic	Reference
Tissue responses at fixed doses	Tissue responses to fixed doses of norepinephrine or acetylcholine	Fleming et al, 1972
EC50	Iris contraction induced by carbachol	Patil, 1993
EC50	Cardiac muscle contraction induced by adrenaline and noradrenaline	Kaumann et al, 1989
Ki and EC50	Ki and EC50 for atrial natriuretic peptide and norepinephrine	Hancock et al, 1988
IC50	Cytotoxic effects of drugs on cancer cells in vitro	Levasseur et al, 1998
Hill coefficient	Electrophysiological response of rat olfactory receptor neurons to odorants	Rospars et al, 2008
Hill coefficient	Force–calcium relationship in myocytes	Walker et al, 2010
K_on_, K_off_, Kd	Antibody-antigen binding	Poulsen et al, 2011
K_on_	Microtubule binding to destabilizing kinesin-8 (Kif18B)	Shrestha et al, 2023
Exponential time constants	Single-molecule transport into liposomes, by a neurotransmitter: sodium symporter	Fitzgerald et al, 2006

**Table 2:** Pharmacokinetic parameters reported to follow lognormal distributions

Parameter	Parameter/Topic	Reference
Clearance, Vd, t_1/2_	Clearance, volume of distribution, and elimination half-life of theophylline after IV infusion	Steinijans et al, 1982; Julious and Debarnot, 2000
Cmax	Either lognormal (Lacey et al) or not (Shen et al)	Lacey et al, 1997; Shen et al, 2017
AUC	Area under the curve (AUC) is close to lognormal	Shen et al, 2017
Whole-body uptake	Post-Chernobyl nuclear accident, whole-body counts of Cs-137 in women	Wertelecki et al, 2016
Plasma levels	Strontium plasma concentrations after oral administration to human volunteers	Li et al, 2006
Drug concentration	Intracellular drug concentration in tumors	Zanotti-Fregonara and Hindié, 2011

Although some papers (and our simulations; see below) report that Hill Slopes are lognormal, Levasseur et al (1998) reported that Hill slopes follow a normal distribution. We digitized their published frequency distribution and fit it using Poisson weighting (because all the values are counts). We found that a lognormal distribution fits somewhat better (pseudo *R*^2^ = 0.84) than a normal distribution (pseudo *R*^2^ = 0.76).

#### 2 Simulations demonstrating pharmacological parameters are lognormal

De Lean et al (1982) demonstrated that Kd values from simulated repeated experiments are closer to lognormal than normal. Christopoulos (1998) used simulations to demonstrate that the distributions of these parameters are lognormal: EC50, Hill coefficient, agonist efficacy *τ* in the Black and Leff operational model of agonism (Black et al, 2010), the receptor/G-protein dissociation equilibrium constant K_G_, and the cooperativity factor *α*. He found that the dissociation rate constant (K_off_) was fit reasonably well by both normal and lognormal distributions and concluded that K_off_ is normal. But his data demonstrates that the lognormal distribution fits those values better than does the normal distribution (the sum-of-squares was smaller for the lognormal fit, and the skewness of the log-transformed data was closer to zero than the skewness of the raw data).

We did similar simulations in more depth assessing the distribution of EC50, Kd, K_off_, K_on_, and Hill coefficient (Supplemental Material). A lognormal distribution fitted all these parameter distributions better (larger *R*^2^) than a normal distribution did. The difference between the corrected Akaike information criteria assesses how much better the data supports one model versus another. If the difference is > 10, the worse-fitting model has essentially no support from the data (Burnham and Anderson, 2002; Portet, 2020). For our simulations, the smallest difference in corrected Akaike information criteria was 30, demonstrating the simulated data for all the parameters support a lognormal distribution substantially better than a normal distribution.

### G Variables defined as the ratio of 2 lognormal distributions are lognormal

#### 1 An interesting and impactful property of lognormal distributions

What is the distribution of a variable defined as the sum, difference, product, or ratio of 2 independent variables? It depends on whether those variables are normal or lognormal.

• The difference or sum of independent normal variables is normal.
• The ratio or product of normal variables is neither normal nor lognormal.
• The difference or sum of lognormal variables is neither normal nor lognormal.
• The ratio or product of independent lognormal variables is lognormal (Limpert et al, 2001). As the next section will show, this relationship has particular importance in pharmacology, where many key parameters arise as ratios of lognormal variables.

#### 2 Examples in pharmacology where important parameters are the ratio of 2 lognormal variables

This relationship has ramifications in pharmacology because many parameters are defined as the ratio of lognormal parameters. Here are examples:

• Bioequivalence. The comparison of drug formulations relies on the ratio of areas under the curve between test and reference formulations. Area under the curve values tend to be lognormally distributed because of the multiplicative nature of drug absorption and elimination processes (https://www.fda.gov/media/70958/download). Consequently, their ratio is lognormal, which dictates the statistical approaches used in bioequivalence studies (Julious, 2004).
• Equilibrium dissociation constant (Kd). The Kd represents the ratio of unbinding to binding rate constants (K_off_/K_on_). Both rate constants arise from multiple molecular steps in protein-ligand interactions that combine multiplicatively, leading to lognormal distributions. Their ratio, Kd, is therefore lognormal, impacting how we analyze drug-receptor binding data.
• The operational model. The transducer ratio *τ* is defined as the total receptor density (termed R_0_ or R_t_) divided by the coupling efficiency constant *K*_E_ (Black and Leff, 1983; Kenakin et al, 2012). Both parameters become lognormally distributed through multiplicative cellular processes—RT through receptor expression and trafficking, *K*_E_ through sequential steps in signal transduction. Consequently, their ratio *τ* is lognormal.
• Biased agonism. Ligand bias is quantified as the transduction coefficients (*τ*/*K*_A_) for different signaling pathways. Because *τ*/*K*_A_ represents a ratio of lognormally distributed parameters, it is itself lognormal. When comparing 2 pathways (eg, G-protein versus *β*-arrestin signaling), the standard approach calculates the difference between log-transformed values for a test ligand [Δlog(*τ*/*K*_A_)] and normalizes this to a reference ligand, yielding ΔΔlog(*τ*/*K*_A_) (Kenakin et al, 2012). Because the log-transformed transduction coefficients for individual pathways should be normally distributed, their differences (Δlog and ΔΔlog values) will also be normal assuming that the pathways respond independently—a reasonable assumption given our understanding of distinct signaling mechanisms. If the signaling pathways are not independent, then a modified operational model has been proposed (Zhu et al, 2019).
• Allosteric interactions. The cooperativity factor *α* is defined as a ratio of binding constants—specifically, the ratio of a ligand’s binding affinity (Ki) when the allosteric site is empty to its affinity when that site is occupied by a modulator (Ki(free)/Ki(co-bound)). Because both Ki values arise from binding processes and are lognormally distributed, their ratio *α* is also lognormal (Christopoulos, 1998; Christopoulos and Kenakin, 2002).

#### 3 Why is the ratio of 2 lognormal variables lognormal?

Consider 2 lognormal variables *X* and *Y*. To understand the distribution of their ratio *Z* = *X*/*Y*, we can use a powerful mathematical tool: taking logarithms transforms multiplicative relationships into additive ones. Take the logarithm of both sides: log(*Z*) = log(*X*) − log(*Y*). Because *X* and *Y* are lognormal, both log(*X*) and log(*Y*) are normal by definition. When we subtract 2 independent normal variables, the result is also normal. The final step follows directly from the definition of a lognormal variable: because log(*Z*) is normal, *Z* itself must be lognormal.

## IV Descriptive statistics of lognormal distributions

A normal distribution is defined by 2 parameters: the mean (ie, arithmetic mean or AMean) and the SD (ie, again, arithmetic). As mentioned earlier, if a distribution is lognormal, then the logarithm of that distribution is normal. Statisticians (and some biomedical scientists) sometimes report the mean and SD of the log-transformed data. When interpreting such data, pay attention to whether common or natural logarithms were used.

Rather than reporting the summary statistics of the set of logarithms, we prefer to use the GeoMean and the geometric SD (GeoSD).

### A The geometric mean

#### 1 The GeoMean of an ideal lognormal distribution or population

**Fig. 4:**
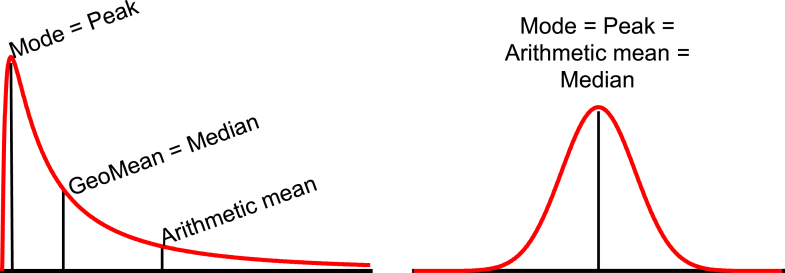
The arithmetic mean, GeoMean, and median of an ideal lognormal distribution. Left: An ideal lognormal distribution showing distinct mode, arithmetic mean, and median (GeoMean). The GeoMean is the median; half the values are larger, and half are smaller. The arithmetic mean is the center of gravity of the distribution. If you made the distribution out of wood or plastic and included the tail that goes far beyond the right limit of the graph, it would balance at the arithmetic mean. Right: An ideal normal distribution showing that the mode, arithmetic mean, and median are identical.

The geometric mean, which we abbreviate GeoMean, is another name for the median of an ideal lognormal distribution. It is expressed in the same units as the data. Figure 4 shows that the GeoMean, mode and AMean have different values for an ideal lognormal distribution (left), but all are identical for an ideal normal distribution (right).

**Fig. 5:**
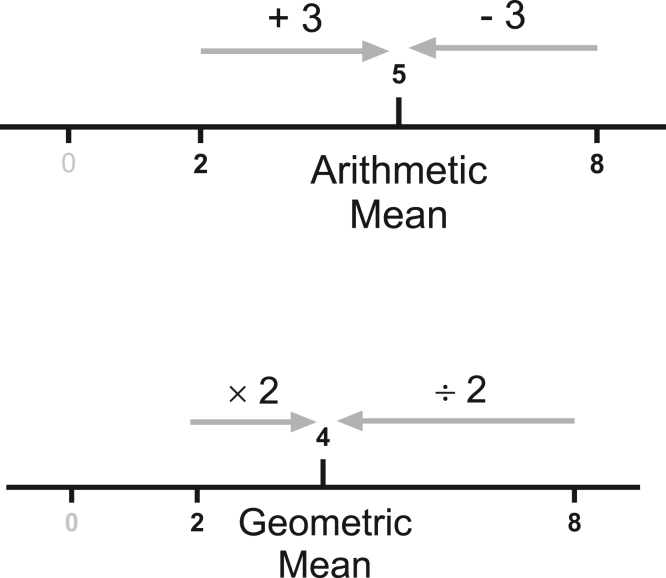
The concept of different kinds of means. What is the mean of 2 and 8? The upper panel shows that the arithmetic mean is 5 because the same delta takes you from that geometric mean to each value (add 3, or subtract 3). The lower panel shows that the geometric mean is 4 because the same multiplicative factor takes you from that geometric mean to each value (multiply by 2, or divide by 2).

The concept of an AMean has been drilled into us since primary school, and it may be hard to imagine how it is possible to have another kind of mean. Figure 5 shows one way to understand how there can be 2 distinct means.

#### 2 How to calculate the GeoMean of a data set

To calculate a GeoMean, first calculate the common (ie, base 10) logarithms of all the values, compute their mean (call it *m*), and then calculate GeoMean = 10^*m*^.

Here, we use base 10 logarithms and the corresponding 10ˆ antilog function, as this is what most biologists are familiar with. Many statisticians, engineers, and physical scientists prefer the natural log (ln) and the corresponding exp() antilog function. The GeoMean (and the GeoSD) will be the same with either approach as long as the same base is used for taking the logarithms and reversing that transform (antilog).

You will sometimes see the GeoMean defined as the *n*-th root of the product of all values. As long as all values are positive, these 2 definitions are equivalent.

#### 3 Relationship between the GeoMean and the median

As mentioned, for an ideal lognormal distribution, the GeoMean and median are identical.

For any particular data set randomly sampled from a lognormal distribution, the obtained GeoMean is equally likely to be larger or smaller than the median. On average, the GeoMean is a more accurate estimate of the population median than is the median computed from the same sample (Parkin, 1993; Vogel, 2022). That is why the GeoMean is the standard way to quantify the center of a lognormal distribution. However, the GeoMean can be a poor estimate of the median if data are sampled from a skewed distribution that is not lognormal, or when data are sampled from a (mostly) lognormal distribution with outliers (Vogel, 2022).

### B The geometric standard deviation

#### 1 What is the GeoSD?

**Fig. 6:**
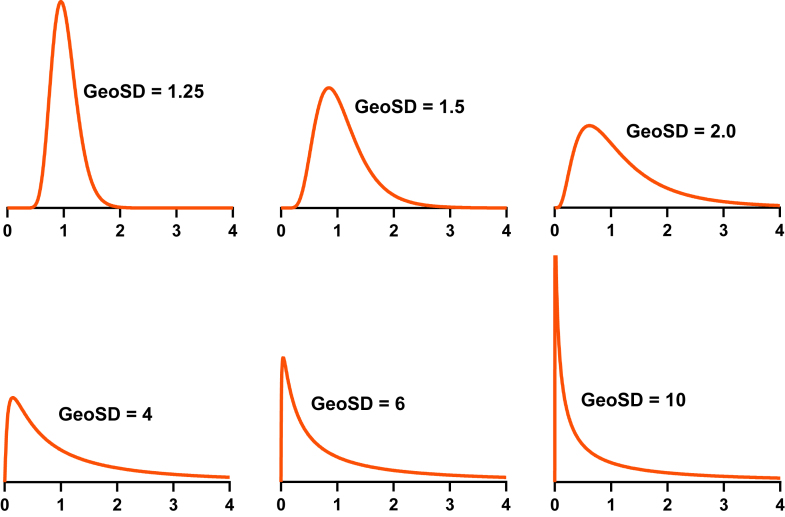
The GeoSD quantifies asymmetry. All the graphs share the same area under the curves (1.0) and same GeoMean (1.0). Lognormal distributions with larger GeoSDs are more skewed.

The GeoSD (Kirkwood, 1979) quantifies both the spread and asymmetry of a lognormal distribution, as shown in Fig. 6. Unlike the arithmetic (regular) SD, which has the same units as the data, the GeoSD has no units. Multiplying the raw data by 1000, say, would make the arithmetic SD commensurately 1000 times larger, but would not change the GeoSD (Lewontin, 1966). The GeoSD always has a value ≥ 1.0. The GeoSD = 1.0 only when all values are identical.

#### 2 Calculating the GeoSD

The GeoSD is calculated as $GeoSD=10^{s}$ where *s* is the SD of the common logarithms of the values in the sample. Equivalently, $GeoSD=e^{s}$, where *s* is the SD of the natural logarithms of the values. Note that your choice to use natural or common logarithms will alter the value of *s* but not GeoSD.

The terms *Geometric SD factor* and *multiplicative SD* are sometimes used as synonyms of GeoSD. Beware of the term *s*∗, as it sometimes serves as an abbreviation for the SD of the natural logarithms, and sometimes for the GeoSD (Limpert et al, 2001).

#### 3 A lognormal distribution with a small GeoSD is nearly identical to a normal distribution

**Fig. 7:**
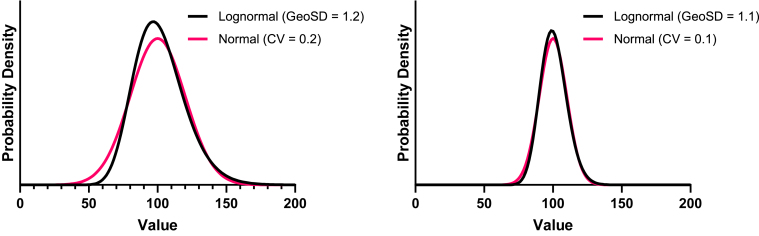
When the CV is less than ∼0.2, normal and lognormal distributions are similar. Left: A normal distribution with mean = 100 and SD = 20 (red), and a lognormal distribution with GeoMean = 100 and GeoSD = 1.2 (blue). Note that the SD is 20% of the mean so the CV, is 20%. Adding 20% is the same as multiplying by 1.20, so the CV of 20% is roughly equivalent to a GeoSD of 1.2. Right: A normal distribution with CV = 0.1 and a lognormal distribution with GeoSD = 1.1.

A lognormal distribution with a small GeoSD closely resembles a normal distribution (Fig. 7). How small does the GeoSD need to be? Any cutoff is somewhat arbitrary, but values of 1.2 (Limpert et al, 2001) and 1.3 (Elassaiss-Schaap and Duisters, 2020) have been suggested.

For variables that are always positive but are approximately normal, variation can be quantified as the coefficient of variation (CV) which equals SD/AMean. A lognormal distribution with a small GeoSD looks very similar to a normal distribution with a CV = GeoSD -1 (Haeckel and Wosniok, 2010). This relationship makes sense because the minimum possible value of the CV is 0.0, and the minimum possible value of the GeoSD is 1.0.

**Fig. 8:**
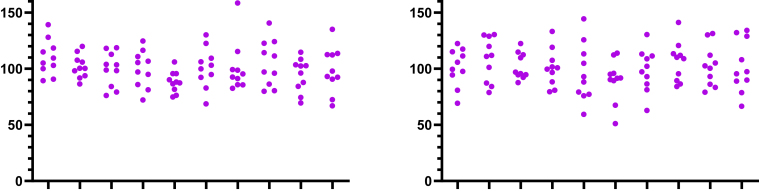
Samples from lognormal vs normal populations. Each panel shows 10 simulated data sets (*n* = 10 each). One was sampled from a lognormal distribution (GeoSD = 1.2; GeoMean = 100), and the other from a normal distribution (SD = 20; mean = 100). You cannot tell which is which by looking at the graphs. (The graph on the left shows lognormal data; the graph on the right shows normal data.)

Figure 8 shows how similar normal and lognormal data can appear. Each panel shows 10 simulated data sets (*n* = 10 each). One was sampled from a lognormal distribution with GeoSD = 1.2 and GeoMean = 100, and the other was sampled from a normal distribution with a SD = 20 and mean = 100 (so the CV = 0.2). It is impossible to guess which is which (in fact, the left panel is lognormal; the right panel is normal).

**Fig. 9:**
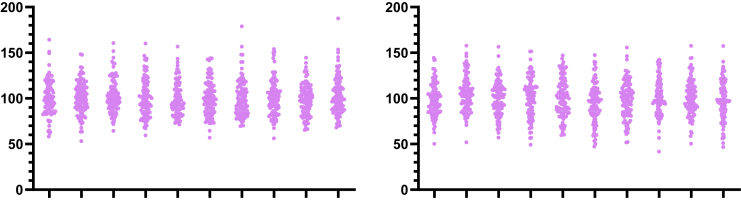
Even with *n* = 100, a normal distribution with CV = 20% is indistinguishable from a lognormal distribution with GeoSD = 2.0. This matches Fig. 8, but with *n* = 100 per data set.

Figure 9 is identical, but with *n* = 100 per group. You still cannot tell which is normal and which is lognormal by inspection.

An example of a variable that fits both normal and lognormal distribution is height. Slavskii et al (2021) reviewed the distribution of height in multiple populations. Its CV is between 0.03 and 0.05, small enough so that normal and lognormal distributions both fit very well (but the lognormal distribution fits slightly better, a finding that is only apparent with huge data sets).

#### 4 How to write the GeoMean and GeoSD

If you assume sampling from a normal distribution, the mean and SD are commonly written as 293.5 ± 515.4 (control data from Fig. 1) and read as “293.5 plus or minus 515.4.” The plus-or-minus symbol is standard. If you assume sampling from a lognormal distribution, the GeoMean and GeoSD would be stated as “103.0 multiplied or divided by 4.53.” This can be written in several ways:

• Enter the multiply × superscripted (in the Insert Symbol dialog of Word) followed by a slash: 103.0 ^×^/ 4.53. This notation was proposed by Limpert and Stahel (2011).
• Enter a superscripted (and bold) period followed by a slash: 103.0 ^.^/ 4.53.
• Insert a multiply sign and a superscripted ±1, for example 103.0 × 4.53^±1^. With the plus sign, this becomes 103.0 × 4.53. With the minus sign, it becomes 103.0 × 4.53^−1^ = 103.0 ÷ 4.53.
• Use the Unicode “DIVISION TIMES” symbol (U+22C7) that superimposes the multiply × and divide ÷ symbols: 103.0 ⋇ 4.53. To enter this character into Microsoft Word, enter “22C7” without the quotation marks, hold the Alt (Windows) or Option (Macs) key, and tap X. Or use the Insert Symbol dialog. GraphPad Prism (starting with version 9) includes this symbol on the Math tab of its Insert Symbol dialog. At the font sizes used in a manuscript, it looks like an asterisk so does not communicate the concept of multiply-or-divide very clearly. At the larger sizes used for presentations, the symbol is more understandable.
• If you want to avoid the phrase “multiplied or divided by,” present a table with separate columns for GeoMean and GeoSD.

### C Other ways to describe lognormal distributions

#### 1 The range that contains 68% or 95% of the values

**Fig. 10:**
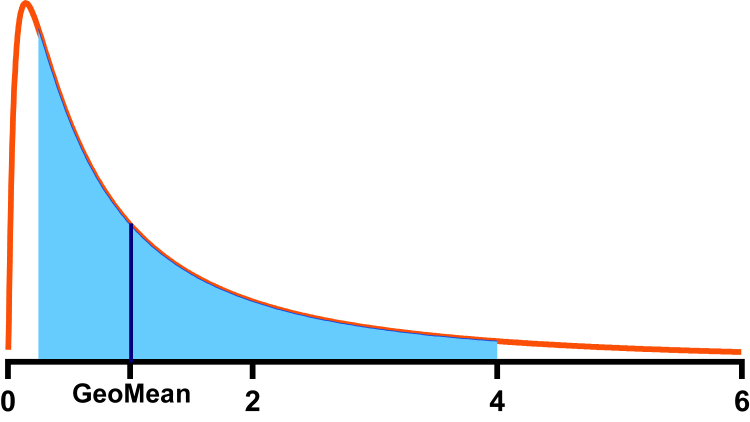
The middle 68.3% of a lognormal distribution. This lognormal distribution has a GeoMean = 1 and a GeoSD = 4. The shaded area extends from the GeoMean divided by the GeoSD to the GeoMean multiplied by the GeoSD. If it looks to you like the shaded area contains > 68% of the total area, that is because the tail of the curve extends far to the right beyond the limits of this graph and that tail has considerable area. In this example, the shaded range does not include the mode, the X-value at the peak of the curve. However, when the GeoSD is much smaller, that range will include the mode.

With a normal distribution, the range of values extending from the [mean − SD] to [mean + SD] includes about two-thirds of the population, more exactly 68.3%. With a lognormal distribution, the comparable range extends from [GeoMean/GeoSD] to [GeoMean × GeoSD] (Fig. 10).

Similarly, about 95% of values of a normal distribution are within the range [mean − 2 SD] to [mean + 2 SD]. The comparable range with a lognormal distribution is [GeoMean / GeoSD^2^] to [GeoMean × GeoSD^2^]. Note that the GeoSD is squared, not doubled.

#### 2 Confidence interval of a GeoMean

To calculate a CI of a GeoMean—perhaps better called a compatibility interval (Rafi and Greenland, 2020)—transform the values to logarithms, compute the CI of the mean for the degree of confidence you want (usually 95%), and then reverse-transform the lower and upper confidence limits using the antilogarithm transform. The resulting interval will not be symmetrical around the GeoMean, but the asymmetry might be subtle.

## V How to decide if a variable is lognormal

Deciding whether to analyze data assuming sampling from lognormal distributions is not always a straightforward process.

### A Do not rely on tests of normality and lognormality

#### 1 Review of normality tests

Normality tests use various criteria to test the null hypothesis that a data set was sampled from a normal distribution. Three commonly used normality tests are those named for D’Agostino–Pearson (D’Agostino et al, 1990), Shapiro–Wilk (Shapiro and Wilk, 1965), and Anderson–Darling (Anderson and Darling, 1954). They use different methods to quantify the discrepancy between the actual data distribution and an ideal normal distribution, so can produce different results. A small *P* value means that sampling from a normal distribution would rarely create a data distribution that far (or further) from normal.

#### 2 How lognormality tests work

To test for lognormality, first transform all the data to their logarithms (it does not matter if you use common or natural logarithms). If the data were sampled from a lognormal distribution, that set of logarithms would have been sampled from a normal distribution. Test this with one or more normality tests. If the *P* value is small, you will conclude that the distribution of the logarithms would be unlikely if the underlying distribution is normal, so the distribution of the original values would be unlikely if the underlying distribution is *lognormal*.

#### 3 Normality and lognormality tests are often inconclusive

Let us revisit the data sets of Fig. 8 where normal and lognormal distributions appeared nearly identical. In the left panel of lognormal data (GeoSD = 1.2), 9 of the 10 data sets pass 3 different normality tests (Anderson–Darling, D’Agostino–Pearson, and Shapiro–Wilk) with *P* > .05, and also pass 3 different lognormality tests (the same tests run on the logarithms of the values) with *P* > .05. The right panel of Fig. 8 shows normal data. All 10 data sets pass the 3 normality tests, and 9 pass the 3 lognormality tests.

**Fig. 11:**
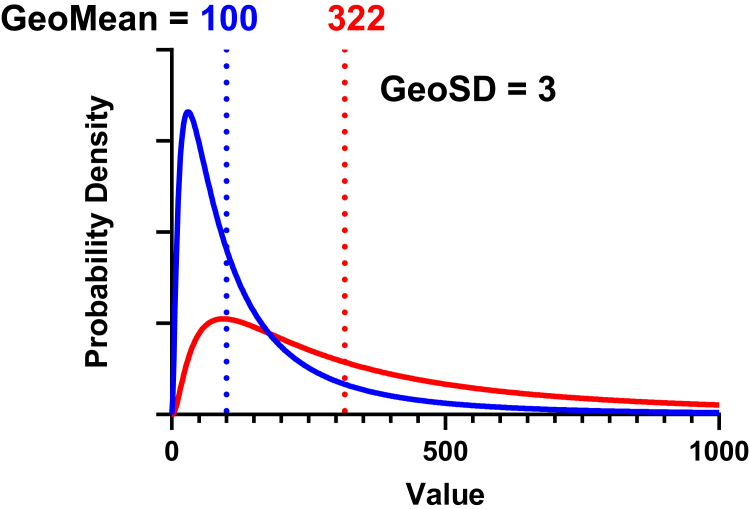
Distributions used to sample data in Figs. 12 and 13. Both lognormal distributions have GeoSD = 3. The GeoMeans differ by a factor of 3.2.

**Fig. 12:**
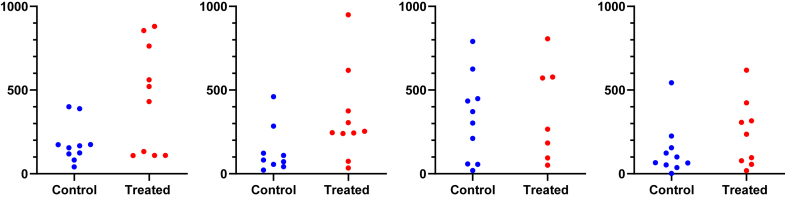
Random samples (*n* = 10) from lognormal distributions shown in Fig. 11.

**Fig. 13:**
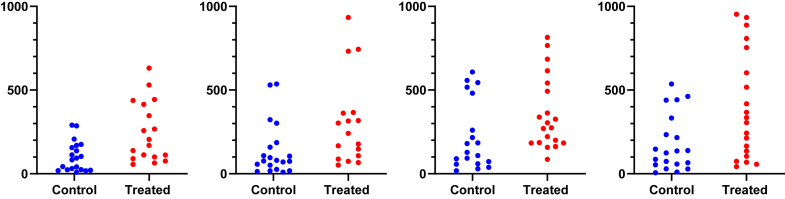
Same as Fig. 12, but with *n* = 20 per sample.

What happens with a larger GeoSD? Figure 11 shows 2 lognormal distributions with different GeoMeans (100 and 322) and identical GeoSD (3). With a GeoSD this large, the asymmetry of the distribution is not subtle. The 2 distributions are quite distinct, but overlap considerably. Note that the samples shown in Figs. 12 and 13 were randomly drawn from these distributions.

Figure 12 shows 4 simulated experiments of data from these distributions with *n* = 10 per group. It is not always obvious by inspection that the data are not normal.

Normality tests cannot reliably determine whether these distributions are normal or lognormal. We ran 3 different normality tests (D’Agostino–Pearson, Shapiro–Wilk, and Anderson–Darling) on 2500 simulated control data sets (*n* = 10) similar to those in Fig. 12. The simulated data sets failed the normality tests a bit more than half of the time (53%, 64%, and 66% for the 3 control sets; and 52%, 63%, and 65% for the treated data sets). In other words, nearly half of the simulated lognormal data sets passed normality tests.

Because the data are sampled from lognormal distributions, you would expect 5% of the simulated data sets to fail the lognormality tests with *α* set to 0.05, and indeed between 4.5% and 5.6% of the simulated data sets failed each of the 3 tests.

Normality tests can better detect the lack of normality of lognormal data when the sample size is larger. Figure 13 doubles the sample size to 20 per group. Now, between 84% and 96% of the simulated data sets (2 treatments; 3 normality tests; 10,000 simulations) fail the normality tests with *P* < .05.

### B How to approach questions about lognormality

We have shown that normality tests can lead to inconsistent and even incorrect conclusions, especially with small data sets. So how should scientists decide when to assume lognormality? Here is the approach we recommend:

#### 1 Ask the right questions

When asking whether a variable is sampled from a lognormal distribution, be sure to frame your question appropriately.

• Recall that the normality assumption that forms the basis of *t* tests, ANOVA, etc, does not refer to the data from a particular experiment; rather it refers to the overall “invisible” distribution (or population) of data from which your experiment is just one sample. Do not ask about the distribution of a particular data set. Any particular data set cannot *be* lognormal (or normal). It only makes sense to ask whether it is reasonable to assume the data were *sampled from* a lognormal (or normal) population or distribution.
• Ask about more than data collected in one particular experiment. Decide how to analyze all experiments measuring a particular variable. The assumption about the distribution of values will apply to all similar experiments with that variable.
• Do not ask if the underlying distribution is *exactly* lognormal (or exactly normal). Ask about *approximate* agreement with the lognormal (or normal) ideal. Why approximate? One reason is that lognormal distributions extend up to infinity, but biological variables have physical limits so their actual distribution cannot extend to infinity. This is also the case for normal distributions, which also extend down to negative infinity. Also note that if the model that generates lognormal distributions is not exactly correct, the distribution may not be exactly lognormal. For example, a combination of additive and multiplicative factors might lead to a distribution that is only approximately lognormal.
• As mentioned earlier, only a ratio scale variable can be lognormal. When considering whether a variable can be lognormal, review this checklist of properties that define ratio scale variables (discussed in the section Ratio scale variables):
  ✓ Negative values must be inconceivable. Lognormal variables can only be positive values.
  ✓ Zero must either mean none of that variable or be the asymptotic value the variable approaches. However, no values can actually equal 0.0.
  ✓ Converting between units must require only multiplication or division.
  ✓ It must make scientific sense to calculate a ratio of 2 values.

If a variable fails to meet any of these criteria, it is not a ratio variable and so cannot be lognormal.

#### 2 Consider the possibility that your data may follow a distribution that resembles lognormal

When working with positively skewed data, beware of other distributions that can resemble the lognormal distribution. Two such distributions are the *γ* distribution and the distribution of the ratio of 2 normally distributed variables.

##### a The γ distribution resembles the lognormal distribution

The *γ* family of probability distributions (Thom, 1958; https://towardsdatascience.com/gamma-distribution-intuition-derivation-and-examples-55f407423840) is commonly used to model waiting times until a certain number of events occur. For example, it can be used to model the time until a specific number of radioactive decay events are observed as well as the time to onset of drug effects.

**Fig. 14:**
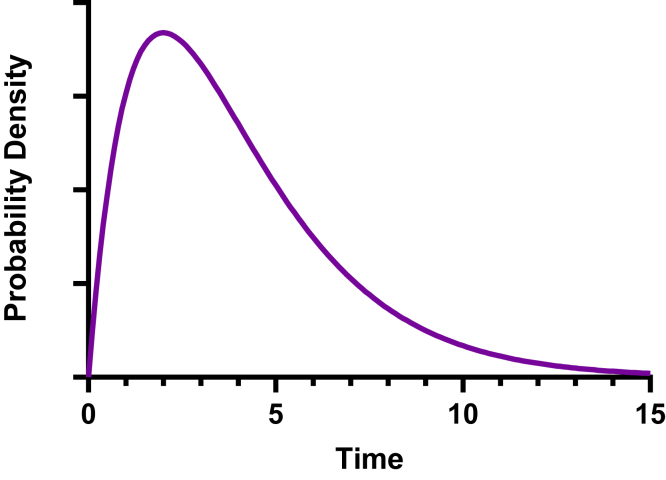
A gamma distribution looks similar to a lognormal distribution. Equation where *X* is time (or duration), and *k* and theta are the 2 parameters that define the distribution (here, both are set to 2.0). *Y* = (1/(gamma(*k*) ∗ thetaˆk)) ∗ *X*ˆ(^k^-^1)^ ∗ exp(-*X*/theta).

Similar to the lognormal distribution, the *γ* distribution is defined only for positive values, is skewed with a long right tail, and is defined by 2 parameters (the average event frequency and the number of events you are waiting for). With certain sets of parameters, a *γ* distribution and a lognormal distribution can look nearly identical (Fig. 14). But it is uncommon for times or durations to have a lognormal distribution, and it hard to imagine any variable other than time or duration having a *γ* distribution.

##### b The distribution of the ratio of 2 normal distributions resembles a lognormal distribution

**Fig. 15:**
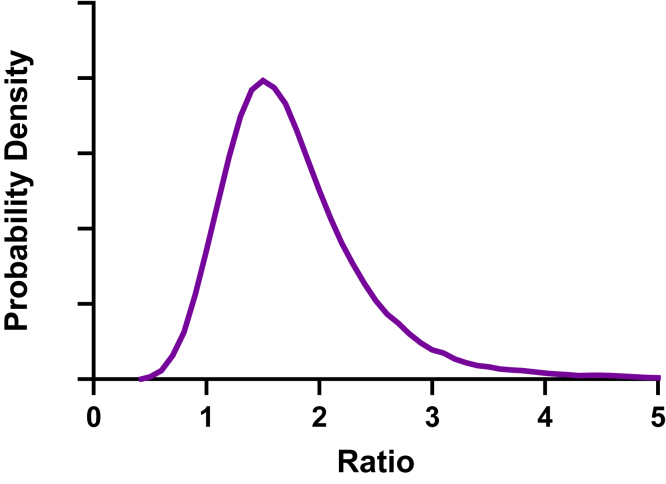
The distribution of the ratio of 2 normal distributions can look similar to a lognormal distribution. The graph shows the smoothed frequency distribution of 10,000 ratios calculated with the numerator drawn from a normal distribution with mean = 100 and SD = 20, and the denominator drawn from a normal distribution with mean = 60 and SD = 15.

The distribution of the ratio of 2 normally distributed variables can be skewed (Fig. 15). This skewness is partly because values in the denominator can be close to zero. Although this skewed shape resembles a lognormal distribution, it is distinct and not lognormal.

### C What to do when you are unsure about lognormality

#### 1 Do not get fooled by variables that are already log transformed

Beware of variables where the log transformation was already done during data collection. One example is acidity. Instruments do not directly report the concentration of hydrogen ions, but rather report the pH (which is −1 times the log_10_ of hydrogen ion concentration in molar). Other examples are the amplitude of earthquakes expressed on the Richter scale (the logarithm of amplitude) and drug potencies as pEC50 (−1 times the logarithm of EC50). With these data, the underlying variable (eg, concentration of [H+], amplitude of earthquake, or EC50) is often lognormal, but the data at hand are already log-transformed (to pH, decibels, or pEC50) so are usually normal.

#### 2 Compare the consistency of the SD versus the consistency of the CV

When assessing if a variable is lognormal, do not just consider one data set at a time. Look for consistency among multiple data sets of the same variable.

**Fig. 16:**
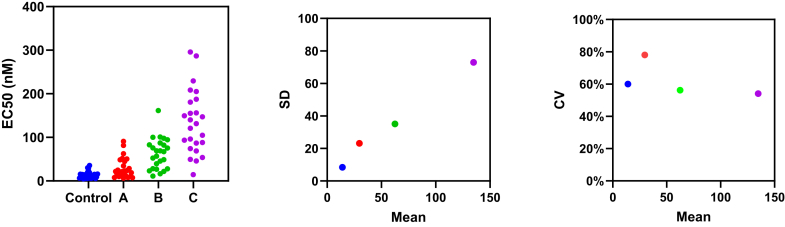
With lognormal distributions, the SDs are proportional to the mean. Left: Four data sets. Middle: The SDs are proportional to the mean. Right: The coefficient of variation (= SD/mean) is consistent for all 4 data sets. This is a clue that the data may be lognormal. In fact, the data were simulated from lognormal distributions with GeoSD = 2.0 and various GeoMeans.

The *t* test and ANOVA assume that the data sets are sampled from normal distributions with the same SD. In contrast, if all the data sets are sampled from lognormal distributions with the same GeoSD, the SDs will differ and be approximately proportional to the AMeans. In other words, when sampling from lognormal distributions with the same GeoSD, you expect the CVs (defined as SD/AMean) to have similar values, but the SDs to have different values (Limpert et al, 2001). Figure 16 shows an example. This method has been used to demonstrate that height is lognormal, not normal (Slavskii et al, 2021).

#### 3 Calculate the likelihood ratio of sampling from normal versus lognormal distributions

What is the relative likelihood of a particular data set being sampled from a normal distribution versus from a lognormal distribution? That likelihood ratio (LR) can be calculated by comparing the fit of the normal and lognormal distributions to a data set. Burnham and Anderson (2002) showed how to calculate the LR, and we reduce the math to a simple equation derived in a Supplemental Material:

$$\text{LR}={\left[\frac{GeoMean}{AMean}\cdot \frac{\ln\left(GeoSD\right)}{CV}\right]}^{n}$$

An LR > 1 means that the data are more likely to have been sampled from a normal distribution, whereas an LR < 1 means that the data are more likely to have been sampled from a lognormal distribution. For example, let us calculate the LR for the control data of Fig. 1. AMean = 293.5, GeoMean = 103, GeoSD = 4.53, CV = 1.76, and *n* = 20. Using the equation above, LR = 3.9 × 10^−11^, demonstrating that the data are overwhelmingly more likely to have been sampled from a lognormal distribution than from a normal distribution.

#### 4 Consider the value of the CV

If a variable can only have positive values, you can learn a bit by thinking about its CV, which is the ratio of the SD divided by the mean. Because the SD and mean are in the same units, the CV is a unitless ratio.

##### a If the CV is small

We have already seen (see Figs. 8 and 9) that when the CV is lower than about 0.2, normal and lognormal distributions are very similar.

##### b If the CV is large

All normal distributions span from negative infinity to positive infinity, which means that some values are negative. However, many normal distributions are almost entirely positive, with only a small negative tail that can be disregarded. After all, assumptions about the ideal distribution of data are, at best, only approximations.

**Fig. 17:**
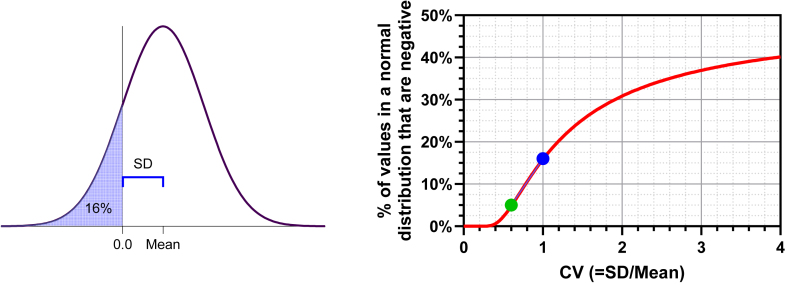
A normal distribution with a high coefficient of variance (CV) contains a substantial number of negative values. Left: If the CV (which equals SD/mean) equals 1.0, then 16% of the values in a normal distribution are negative. Right: The percentage of negative values in a normal distribution as a function of the CV calculated with this equation: 100 × (1-zdist (1/CV)). The blue dot shows that when CV = 1, 16% of the values are negative. The green dot shows that when CV = 0.6, only 5% of the values are negative. Equation: 100 × (1-zdist (1/CV)) (Prism format), 100 × NORM.S.DIST (−1/CV, TRUE) (Excel format), or 100 × pnorm (−1/CV) (R format).

The proportion of a normal distribution that is negative is a function of the CV. The left panel of Fig. 17 displays a normal distribution with a CV = 1.0, meaning that the mean equals the SD. In a normal distribution, approximately 68% of the values fall within 1 SD of the mean. Therefore, if the SD equals the mean, about 16% (= (100% − 68%)/2) of the values are negative (see Fig. 17, left panel). The right panel illustrates the relationship between the CV and the fraction of a normal distribution that is negative. The blue dot represents CV = 1.0, indicating that 16% of the values are negative, whereas the green dot represents CV = 0.6, showing that 5% of the values are negative.

If a variable can only take on positive values, a CV greater than approximately 0.6 tells you that the data were probably not sampled from a normal distribution, even as an approximation. This is because in a typical sample, a nontrivial portion of the values would be negative if the distribution were normal. Limpert and Stahel (2011) point out that it is common to see published papers where data are analyzed as if normal even though this rule of thumb is violated.

#### 5 Do not base your decision only on normality and lognormality tests

Many scientists, we suspect, use the rule: Assume data are normal until proven otherwise. Lognormality, for these scientists, needs to be proven. In other words, “let the data decide.” Keene (1995) presents 3 reasons why this is a bad idea (and we agree):

• As we have seen in Figs. 8 and 9, the results of normality and lognormality tests can be ambiguous. You may want to let the data decide, but analyses of the data do not always lead to a clear decision. Many data sets pass both normality and lognormality.
• Although it might seem logical to first run one test (here the normality test) and use that result to decide what to do next (whether to log-transform the data), this kind of 2-stage approach to statistical testing can lead to misleading results so is not recommended (Cartwright, 1991; Zimmerman, 1996, 2004; Rochon et al, 2012; Gelman and Loken, 2014; Delacre et al, 2017; Shamsudheen and Hennig, 2023).
• If you do multiple similar experiments, you might end up making different decisions for different experiments or even different parts of the same experiment. It is better to analyze all similar data using the same assumptions. This is because the lognormality (or normality) assumption refers to the underlying population, not to a particular sample.

These problems, of course, are intrinsic to the difficulty in distinguishing normal from lognormal distributions, not just running normality tests. You will encounter the same issue by inspecting frequency distributions or quantile–quantile plots.

### D Our recommendation: Choose to assume lognormality based on the nature of the variable without normality testing

We strongly urge scientists to assume data are lognormal largely based on the nature of the variable (or parameter) being compared, and not to rely on normality or lognormality testing. This may sound like extreme advice far from the consensus, but others have given the same advice:

• “The theoretical justification for using this [the logarithmic] transformation for most scientific observations is probably better than that for using no transformation at all… …If it were the normal custom, when scientific observations show uncontrolled variations large compared with the observations themselves, to convert them to logarithms before estimating their mean or variance, the usual result would be an increase in the accuracy and scope of the conclusions drawn from them” (Gaddum, 1945).
• “I suggest that when an a priori decision about distribution has to be made, the lognormal distribution should always be preferred over the normal distribution for data of this general type” (referring to variables, such as concentrations, that can only have positive values) (Heath, 1967).
• “It is recommended that log transformed analyses should frequently be preferred to untransformed analyses, and that careful consideration should be given to use of a log transformation at the protocol design stage. … If the use of a log transformation is chosen on a case-by-case basis, then this will lead to inconsistencies and sometimes the wrong choice will be made.” (Keene, 1995)
• “It is proposed that all quantities should be considered to be lognormal in clinical chemistry if the type of distribution is unknown. Then, laboratories need not decide whether a distribution is quasi-Gaussian or non-Gaussian.” (Haeckel and Wosniok, 2010)
• “The lognormal distribution should be the first choice when modeling data taking (only) positive values. Its empirical as well as theoretical justification is much stronger than for the normal distribution.” (Limpert and Stahel, 2017)
• “You should (usually) log transform your positive data. The reason for log transforming your data is not to deal with skewness or to get closer to a normal distribution…The reason for log transformation is in many settings it should make additive and linear models make more sense. A multiplicative model on the original scale corresponds to an additive model on the log scale. For example, a treatment that increases prices by 2%, rather than a treatment that increases prices by $20. The log transformation is particularly relevant when the data vary a lot on the relative scale. Increasing prices by 2% has a much different dollar effect for a $10 item than a $1000 item” (A. Gelman 2019; https://statmodeling.stat.columbia.edu/2019/08/21/you-should-usually-log-transform-your-positive-data/).

## VI Do not use standard outlier tests with lognormal data

### A Review of outlier tests

Most outlier tests evaluate whether extreme values are likely to have come from a normal distribution. These tests compute the probability that a value as extreme (or more extreme) as the one observed would occur by chance if the data were sampled from a normal distribution. If the *P* value is small (usually < .05), that extreme value is identified as an “outlier,” and some investigators in some situations will remove that value from further analyses (or might report results with and without the outlier).

In a set of many samples from a normal distribution, the most extreme value in 5% of those samples will be identified as an outlier. Note that the 5% probability refers to the fraction of *samples* where the largest value is identified as an outlier, not the fraction of *values* that are identified as outliers.

### B Outlier tests on the sample data

When the raw data of Fig. 1 were analyzed using Grubbs’ outlier test, the largest value in the treated group was identified as an outlier as it is far from the rest of the data. But, as the next section demonstrates, this test is very misleading with untransformed lognormal data. After the sample data were log-transformed, Grubbs’ test did not identify an outlier in either group.

### C Outlier tests on lognormal data

**Fig. 18:**
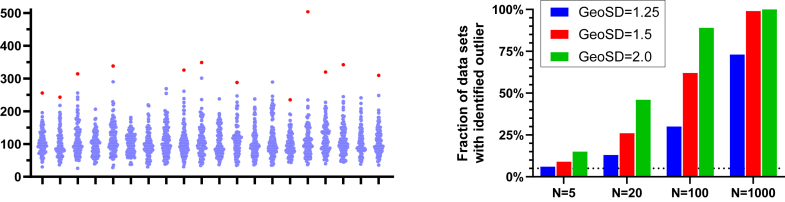
Too many outliers identified with lognormal data. Left: Twenty simulated data sets with *n* = 100, GeoMean =100, and GeoSD = 1.5. The red dots on 12 of the data sets denote outliers identified by Grubbs’ test (*α* = 0.05). Right: Fraction of simulated lognormal data sets where Grubbs’ outlier test detected an outlier with *P* < .05. For each combination of sample size and GeoSD, 1000 data sets were simulated. The horizontal line shows that Grubbs’ test identifies an outlier with *P* < .05 in 5% of data sets sampled from normal distributions (with any sample size).

Standard outlier tests fail dramatically with lognormal data. Because lognormal distributions are naturally skewed, large values that appear to be outliers are actually an expected feature of the distribution. Fig. 18 (left panel) demonstrates this problem using 20 simulated data sets (*n* = 100 each, GeoMean = 100, GeoSD = 1.5). Although many of these data sets look approximately normal, Grubbs’ outlier test (which assumes normality) incorrectly identified the largest value as an “outlier” (*P* < .05) in 12 of the 20 data sets.

The severity of this problem increases with both sample size and GeoSD, as shown in the right panel of Fig. 18. Even with a very modest GeoSD of 1.25—where the distributions look nearly normal—far more than 5% of data sets have their largest value incorrectly flagged as an outlier. With larger sample sizes or larger GeoSD values, nearly every data set has its largest value misidentified as an outlier.

This problem is not limited to formal outlier tests. Visual inspection of data for outliers is equally unreliable with lognormal data, as our eyes are naturally drawn to values that seem “too large” when we expect a symmetric distribution. The key lesson is clear: before testing for or removing outliers, you must first determine whether your data might be lognormal. If the data are lognormal, outlier detection should only be performed after logarithmic transformation.

## VII Comparing 2 groups of lognormal data

### A Lognormal t test assuming sampling from lognormal distributions with equal GeoSDs

#### 1 Calculating the lognormal t test and reporting the results

**Fig. 19:**
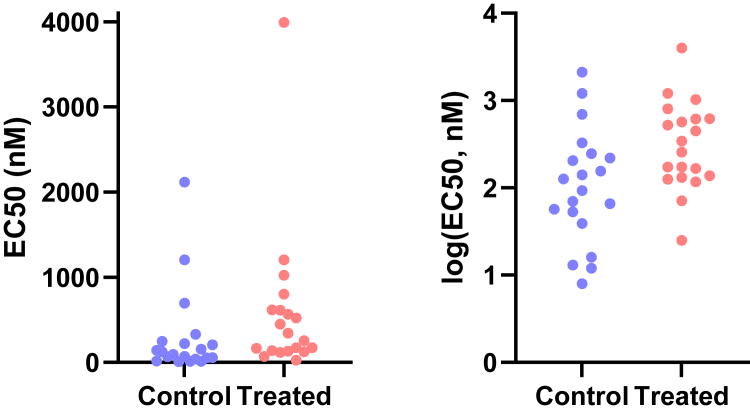
Example data for unpaired *t* test displayed on a linear axis (left) and after being transformed into common logarithms (right). These are the same data as in Fig. 1.

Figure 19 shows example data comparing 2 groups. With lognormal data, it is most common to compare the GeoMeans. For this example, the GeoMean for the control values is 103 nM and the GeoMean for the treated samples is 302 nM. With lognormal data, it rarely (if ever) makes sense to think about the absolute difference between GeoMeans, but instead it makes scientific sense to think about ratios (Wolfe and Carlin, 1999). The ratio is 302/103 = 2.9. In other words, the treatment nearly tripled the EC50.

To calculate a CI and *P* value, we need to run a statistical test. This can easily be done by transforming all values to their logarithms to turn the lognormal distributions into normal distributions, then running an unpaired *t* test on those logarithms. We call this the *lognormal* t *test*. The transformed values are shown on the right panel of Fig. 19. Note that the distribution appears symmetrical, as expected for data that are (before log transforming) sampled from a lognormal distribution. The largest log-transformed value in the treated group is just a bit larger than the rest, and Grubbs’ test on these data does not identify it as an outlier.

The transformed values were analyzed by a 2-sample *t* test using GraphPad Prism 10.4, but any statistical software would give the same result. The difference between the means of the logEC50 values is 0.467. Recall that for any 2 positive values *A* and *B*,

$$\log\left(A\right)-\log\left(B\right)=\log\left(\frac{A}{B}\right),\text{and}\;\text{so}\;10^{\log\left(A\right)-\log\left(B\right)}=\frac{A}{B}\text{.}$$

Therefore 10^0.467^ = 2.9 is the ratio of GeoMeans (as we already determined).

The *t* test reports that the 95% CI for the difference between the means of the logarithms range from 0.09435 to 0.8409. Therefore, the 95% CI for the ratio of GeoMeans ranges from 10^0.09435^ to 10^0.8409^, or 1.24 to 6.93. This gives a good sense of how precisely we have determined the GeoMean ratio.

The above lognormal *t* test reports a 2-sided *P* value of .0154. This *P* value tests the null hypothesis that the 2 sets of logEC50 values are sampled from normal distributions (or populations) that have identical means and SDs. Equivalently, if you refer instead to the EC50s (ie, not logged), then the null hypothesis is that the 2 sets of values are sampled from lognormal distributions with identical GeoMeans and GeoSDs. If the null hypothesis were true, then a ratio of GeoMeans of 2.9 or larger would occur only in around 0.77% of experiments (one tail), and a ratio of 1/2.9 = 0.34 or lower would occur in around 0.77% of experiments (the other tail). Why 0.77%? This is the *P* value (*P* = .0154, ie, 1.54%), divided equally between the lower and upper tails. The *P* value is less than the traditional cutoff (*α*) of .05. Therefore, if you chose that value of *α* and accept all the assumptions of a *t* test, you can reject that null hypothesis.

Note the consistency of the CI and the *P* value. The *P* value is < .05, and the 95% CI of the ratio (1.24–6.93, from above) does not include the value that defines the null hypothesis (1.0).

#### 2 Graphing the results of a lognormal *t* test

**Fig. 20:**
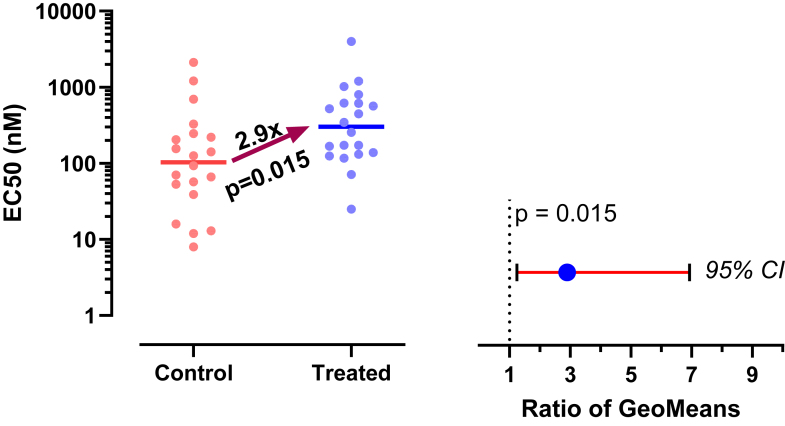
Plotting the results of a 2-sample (unpaired) *t* test of lognormal data. Left: Raw data on logarithmic axis. Right: Ratio of GeoMeans with 95% CI.

**Fig. 21:**
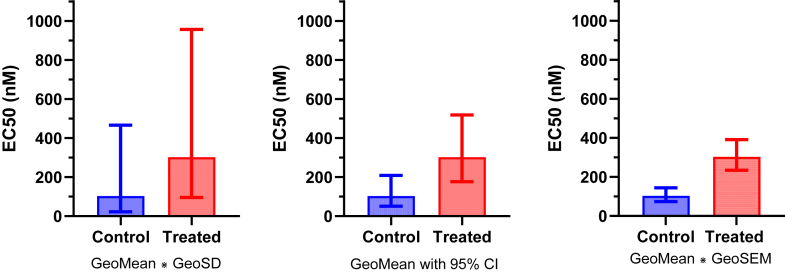
Plotting the results of a 2-sample (unpaired) *t* test of lognormal data with error bars. Left: GeoMean, with error bars showing the GeoMean multiplied or divided by the GeoSD. Middle: Same, but with 95% CI instead. Right: Same as the left panel, but replacing GeoSD with GeoSEM.

Figure 20 shows one way to plot the raw data and results of a lognormal *t* test. If you prefer plotting bar graphs with error bars, Figure 21 shows how. All 3 panels plot the GeoMeans. Lognormal distributions are asymmetrical, so the error bars in all 3 panels are asymmetrical. The error bars on the left show the variation among the values, expressed as the GeoMean divided or multiplied by the GeoSD. The error bars in the middle panel show how precisely the GeoMeans have been determined, expressed as the 95% CI of the GeoMeans. The error bars on the right show the GeoMean multiplied or divided by the geometric standard error (GeoSEM).

**Fig. 22:**
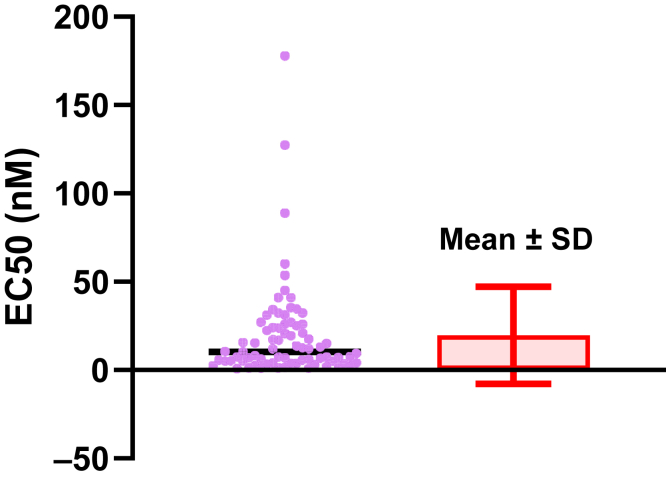
Mean ± SD is not a useful way to quantify variability of lognormal data. Left: Simulated data (*n* = 200, GeoMean = 10, GeoSD = 2.0). Right: Bar graph showing arithmetic mean ± SD. Note that the error bar extends down to negative values, which is impossible with lognormal data. The raw data are in Supplemental Material.

Data are often presented as AMean with error bars representing the SD. If the values were sampled from a normal distribution, this would be straightforward to interpret. The range [mean − SD] to [mean + SD] would contain about two-thirds of the values. But this interpretation does not work when data are sampled from a lognormal distribution (Fig. 22). When the GeoSD is reasonably high so the data are noticeably asymmetrical, the symmetrical ± SD error bars do a poor job of displaying the distribution of the data. In some cases, as shown in this example, the lower error bar can extend downward to a negative value, which makes no sense because lognormal variables can never be negative.

#### 3 Terms to avoid when reporting ratio results

When describing the relationship between 2 geometric means, it is essential to use clear and consistent language to avoid confusion. In the example above, the ratio of geometric means (treated/control) is 2.9. Some scientists would report that value as a ratio, and others would say that the treated response was 2.9 times the control response.

The following terms are ambiguous, and we suggest simply avoiding them:

• *Percent increase (or decrease).* Some might state that the treated GeoMean is 190% higher than that of the control (2.9 × 100% − 100%). But it is also the case that the control GeoMean is 65.5% lower than the treated GeoMean. The 2 are not symmetrical, so we recommend avoiding both. Cole and Altman (2017) define a percentage difference that is symmetrical: 100 × (difference/mean). In our example, the 2 GeoMeans were 103 nM (control) and 302 nM (treated). The average is 202.5 nM, and the difference is 199 nM. The difference/mean is 98% if you compute the increase from control to treated, or −98% if you look at the decrease from treated to control. Although this definition is symmetrical, it is used rarely. We do not recommend it.
• *Fold increase (or decrease).* We agree with Small (2016) that the term *fold increase* should be avoided because it is used inconsistently and so is ambiguous. Some would say there was “a 1.9-fold increase”—the difference between ratios of 1.0 (no change) and 2.9 (observed)—and others would say there was “a 2.9-fold increase” (because the ratio is 2.9). Avoid this confusing term.

### B The lognormal Welch’s *t* test

In the previous example, we considered data sampled from 2 lognormal distributions with different geometric means but equal geometric SDs. Under these conditions, applying an unpaired *t* test to the log-transformed data—referred to as the lognormal *t* test—works well because the 2 transformed distributions have similar variances. However, if the underlying distributions differ not only in their GeoMeans but also in their GeoSDs, the assumption of equal variances after log-transformation is violated. In this scenario, the lognormal *t* test is no longer appropriate, and the log-transformed data should instead be analyzed using the Welch’s *t* test. We refer to this approach as the ***lognormal Welch’s* t *test***.

With the sample data (see Fig. 19), the lognormal Welch’s *t* test reports a *P* value of .016. The means of the logarithms differ by 0.4676 with a 95% CI ranging from 0.09349 to 0.8417. Take the antilog of all 3 values to obtain the ratio of GeoMeans (2.93) and its 95% CI (1.24–6.95). For this example, the lognormal *t* test (prior section) and the lognormal Welch’s *t* test give nearly identical results.

### C Comparing the lognormal *t* test with the lognormal Welch’s *t* test

#### 1 Power

**Fig. 23:**
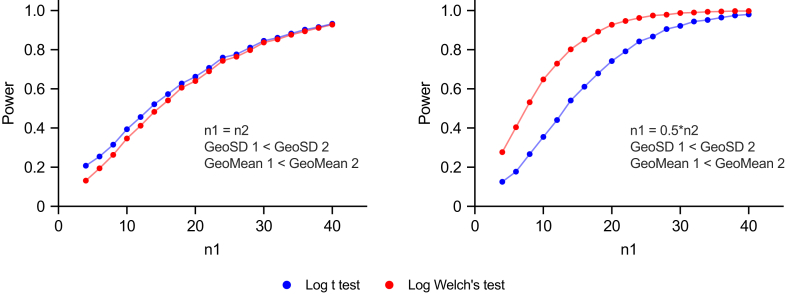
The power of the lognormal *t* test vs lognormal Welch’s *t* test, both applied to lognormal data. For each combination of GeoMean, GeoSD, and sample size, we simulated 2000 experiments with values sampled from lognormal distributions, ran both statistical tests on each simulated data set, and tabulated the fraction of *P* values that are < .05 (our preset *α*). In both panels, we set GeoMean 1 = 1.6, GeoMean 2 = 4.5, GeoSD 1 = 1.2, and GeoSD 2 = 6.0. However, in the right panel, one group is twice as larger as the other. The R code used for these simulations is included in Supplemental Material. Left: The lognormal *t* test (blue) and lognormal Welch’s *t* test (red) have nearly equal power when GeoSD values are unequal, as long as sample sizes are equal. Right: The lognormal Welch’s *t* test has more power when both sample sizes and GeoSD values differ, and when the group with the larger sample size also has a larger GeoSD.

Figure 23 shows the results of simulations comparing the power of the lognormal *t* test with the power of the lognormal Welch’s *t* test, when applied to lognormal data. They have nearly equal power when the sample sizes are equal, even when the GeoSD values are unequal (left panel), but the lognormal Welch’s *t* test has more power when both sample sizes and GeoSD values differ (right panel).

#### 2 Type I error

The type I error rate is the probability of falsely rejecting the null hypothesis. With *α* set to 0.05, a well-behaved test should yield a type I error rate near 5% when its assumptions are met and the null hypothesis is true.

**Fig. 24:**
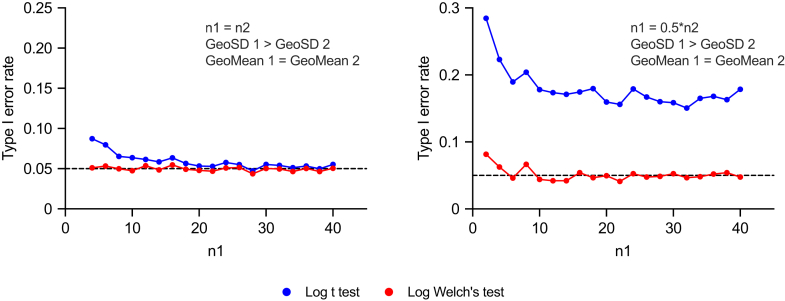
The control of type I error by the lognormal *t* test and the lognormal Welch’s *t* test on lognormal data. For each combination of GeoMean, GeoSD, and sample size, we simulated 2000 experiments sampled from lognormal distributions, ran both statistical tests on each simulated data set, and tabulated how frequently *P* < .05 (our preset *α*). The GeoMeans are the same in both groups, that is, 1.6 (left panel) and 2.7 (right panel), whereas in both panels the GeoSD values differ (ie, GeoSD 1 = 6.0 and GeoSD 2 = 1.22). The R code is included in the Supplemental Material. Left: Both lognormal *t* test (blue) and lognormal Welch’s *t* test (red) control the type I error reasonably well when GeoSD values are unequal, as long as sample sizes are equal. Right: The lognormal Welch’s *t* test controls the type I error much better when both sample sizes and GeoSD values differ.

Although the *t* test is fairly robust to violations of the equal variance assumption when sample sizes are equal, its type I error rate can increase when there are pronounced differences in both variance and sample size (Havlicek and Peterson, 1974; Ramsey, 1980; Posten et al, 1982; Zimmerman, 1987). We confirmed this finding with lognormal data (Fig. 24). Both the lognormal *t* test and the lognormal Welch’s *t* test do a reasonable job when the sample sizes are equal, even when the GeoSD values are unequal (left panel). However, the lognormal Welch’s *t* test did a much better job of controlling type I errors when both sample sizes and GeoSD values differed (right panel).

#### 3 When to use the lognormal Welch’s *t* test

Many researchers recommend using the Welch’s *t* test by default, rather than the traditional unpaired *t* test, regardless of whether variances appear equal (Moser et al, 1989; Rasch et al, 2011; Delacre et al, 2017). Importantly, this choice should be made without first performing a test for equal variances, as doing so can inflate the overall type I error rate (Zimmerman, 2004; Ruxton, 2006; Delacre et al, 2017). Extending this idea to lognormal data, we recommend routinely using the lognormal Welch’s *t* test rather than regular lognormal *t* test. With unequal sample sizes, this choice can matter a lot (see Figs. 23 and 24). With equal sample sizes, the choice matters less, but if there is any possibility that the 2 populations have different GeoSD (that possibility can rarely be ruled out), we recommend using the lognormal Welch’s *t* test.

### D Nonparametric tests with lognormal data

#### 1 Understanding the Mann-Whitney and Brunner-Munzel tests

When analyzing data sampled from lognormal distributions, researchers may notice that the data do not seem to be normal and so consider using a nonparametric test that does not make any assumption about sampling from any specified distribution. However, these nonparametric alternatives come with their own complexities and limitations.

Two nonparametric tests used to compare 2 groups of continuous data are the Brunner-Munzel test (Brunner and Munzel, 2000), and the better-known Mann-Whitney test, also called the Mann-Whitney *U* test. Wilcoxon independently derived an equivalent test, so the names Wilcoxon rank sum test, Mann-Whitney-Wilcoxon test, and Wilcoxon-Mann-Whitney test are also used for this test. (Note: Do not confuse with the *Wilcoxon* *signed-rank* test, which is for analysis of paired data.)

The Brunner-Munzel and Mann-Whitney tests share some fundamental properties. Both analyze the ranks of the values rather than the values themselves and so make no assumptions about the underlying distribution. Because a logarithmic transformation preserves the rankings of positive values, each test gives identical results on raw and log-transformed data. Both are tests of stochastic equality, testing whether values from one group are generally larger (or smaller) than values from the other. More precisely, their null hypothesis is that when randomly selecting one value from each group, there is a 50% chance the larger value came from either group.

The Mann-Whitney test combines values from both groups, ranks them, and compares the average ranks of each group. The Brunner-Munzel test takes a different approach, calculating for each value in group A, the proportion of group B values that are smaller, and vice versa. By combining these proportions, it extracts more information from tied values than the Mann-Whitney test, and it accurately detects differences between distributions even when they have different shapes. This versatility prompted Karch to recommend using the Brunner-Munzel test routinely instead of the Mann-Whitney test (Karch, 2021). While not yet included in most statistical software, the Brunner-Munzel test is available in R, Python, and Jamovi (Karch, 2023).

#### 2 The effect size reported by nonparametric tests

The Mann-Whitney test is often presented as a comparison of medians, which can seem appealing for analyzing data sampled from lognormal distributions. Because the GeoMean of a lognormal sample estimates the median of its underlying distribution, one might be tempted to interpret the Mann-Whitney test as a straightforward comparison of geometric means. Indeed, some implementations of the Mann-Whitney test even report differences between medians with CIs. However, this interpretation is only valid if the 2 distributions are identical in shape and differ only in location (Stonehouse and Forrester, 1998; Fagerland and Sandvik, 2009).

This assumption that both distributions have the same shape does not hold with lognormal data (Divine et al, 2018). Although 2 normal distributions will have the same shape when their SDs are equal, 2 lognormal distributions with different GeoMeans will have different shapes even if they have the same GeoSD. Thus, the assumption required to interpret the Mann-Whitney test as a median (or GeoMean) comparison rarely applies to lognormal data. Moreover, it has been shown that the Mann-Whitney test can yield small *P* values when comparing lognormal distributions with the same GeoMean but different GeoSDs, further complicating its interpretation (Fagerland, 2012).

The results of a Brunner-Munzel test can be summarized as a probability of superiority (with a CI). This is the probability that a randomly selected value from group A will be larger than a randomly selected value from group B. For example, a probability of superiority of 0.8 indicates an 80% chance that a random value from group A exceeds a random value from group B. This is a less intuitive way to summarize the result than the ratio of GeoMeans reported by a lognormal *t* test.

#### 3 The statistical power of nonparametric tests with lognormal data

We ran Monte Carlo simulations to evaluate the power of various statistical tests when analyzing lognormal data. For each experimental design, we specified the GeoMean, GeoSD, and sample size of each group. We simulated 2000 experiments, and tabulated the fraction of *P* values that are < .05 (our preset *α*). The R code used for these simulations is included in Supplemental Material.

**Fig. 25:**
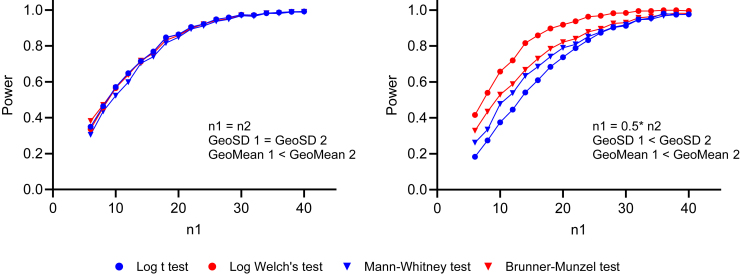
Power of parametric and nonparametric tests applied to lognormal data. For each combination of GeoMean, GeoSD, and sample size, we simulated 2000 experiments, sampling from lognormal distributions, ran 4 statistical tests on each simulated data set, and tabulated the fraction of *P* values that are < .05 (our preset *α*). The R code used for these simulations is included in Supplemental Material. Left: The GeoSDs of the 2 groups were set to the same value (2.7), and the sample sizes were also made equal. The GeoMean of the second group was 2.7 times the GeoMean of the first group (ie, 4.5 vs 1.6). The power of all the lognormal *t* test (blue, circles), lognormal Welch’s *t* test (red, circles), Mann-Whitney test (blue, triangles), and Brunner-Munzel test (red, triangles) were nearly the same. Right: The sample sizes of the first group were set to half the sample size of the second, and the GeoSD values were set to 1.2 and 6.0. Under these conditions, the power of the various tests differs.

When both sample sizes and GeoSDs are equal, the power of the Mann-Whitney and Brunner-Munzel tests is almost identical, and is close to the power of the lognormal *t* test and lognormal Welch’s *t* test (Fig. 25, left panel). However, with unequal sample sizes and GeoSDs, the power of the tests can vary considerably (see Fig. 25, right panel). Under these conditions, the lognormal Welch’s *t* test has the most power. The nonparametric tests have a bit less power, with the Brunner-Munzel test performing better than the Mann-Whitney test. The lognormal *t* test has the least power of the 4 tests.

#### 4 Type I error control

**Fig. 26:**
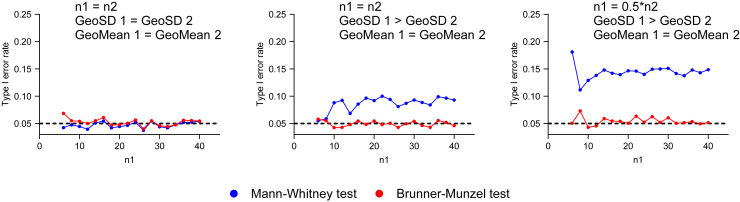
Type I error of parametric and nonparametric tests applied to lognormal data. The method matches that of Fig. 24. In all 3 panels, the GeoMeans of both groups were set to 1.6. Left: The GeoSDs of the 2 groups were set to the same value (6.0), and the sample sizes were also made equal. The type I error Mann-Whitney test (blue), and Brunner-Munzel test (red) were nearly the same. Middle: The GeoSD values were set to 6.0 and 1.2, but the sample sizes were kept equal. Right: The GeoSDs were the same as in the middle panel, but the sample sizes of the second group were set to half the sample size of the first. Under these conditions, the type I error of the Brunner-Munzel was always close to 0.05, but the type I error of the Mann-Whitney test was several fold higher.

Figure 26 compares the type I error rates of the statistical tests in simulated lognormal data. In all these simulations, the GeoMeans were set to the same value. When the distributions had identical GeoSDs and sample sizes (left panel), both nonparametric tests maintained type I error rates near 0.05. However, when the distributions have different GeoSDs (see Fig. 26, middle panel), the type I error rate of the Mann-Whitney test is inflated, as previously observed (Karch, 2021). The inflation of type I error rate for the Mann-Whitney test is increased further when both sample sizes and GeoSDs are set to unequal values (right panel). In contrast, the type I error rate of the Brunner-Munzel test remains near its nominal value of 0.05 for all experimental designs, so long as the sample sizes in both groups are greater than or equal to about 10.

#### 5 Conclusions about nonparametric tests for lognormal data

For analyzing lognormal data, the lognormal Welch’s *t* test is the optimal choice for several reasons. First, it controls the type I error rate, that is, it provides acccurate *P* values when no effect exists. Second, it maximizes statistical power. Third, it provides easily interpretable effect sizes (ratios of GeoMeans), and directly addresses the data’s multiplicative nature. The lognormal *t* test has the same advantages except that when GeoSDs differ, it is less powerful than the lognormal Welch’s *t* test (see Fig. 25).

If you are not sure about the underlying distribution but suspect it might be lognormal, you may prefer to use a nonparametric test. If the experiment has equal sample sizes, both nonparametric tests perform similarly, but the Brunner-Munzel test has a bit more power. If the sample sizes differ substantially, avoid the Mann-Whitney test as it has lower power and larger type I errors than the Brunner-Munzel test. Thus, if you want to use a nonparametric test with lognormal data, choose the Brunner-Munzel test because it has more power and a smaller (more appropriate) type I error.

### E Why the unpaired t test (without log transformation) should be avoided with lognormal data

#### 1 Results of analyzing the sample data with an unpaired t test without log transformation

Let us return to our motivating example (see Fig. 1). The unpaired *t* test (without log transformation) yielded *P* = .22, so the null hypothesis could not be rejected. The 95% CI for the difference between means ranged from −175 to 739. With such a wide CI, the data are consistent with no difference, a moderate decrease, or a large increase. In other words, no conclusion is possible.

#### 2 Problems when analyzing lognormal data as normal

##### a Less useful effect size (difference, rather than ratio)

When you run a *t* test assuming sampling from normal distributions, the effect size is reported most simply as the *difference* between the 2 means. With ratio variables, the difference is rarely a useful way to view the effect, so many scientists do not bother reporting that difference or its CI.

When you run a *t* test assuming sampling from lognormal distributions, the analogous effect size is reported as the *ratio* of the 2 GeoMeans. A ratio is a logical way to think about the size of an experimental effect. And it is helpful to report the ratio with its CI to give a sense of how precisely the ratio has been determined.

##### b Loss of statistical power (for a given sample size)

**Fig. 27:**
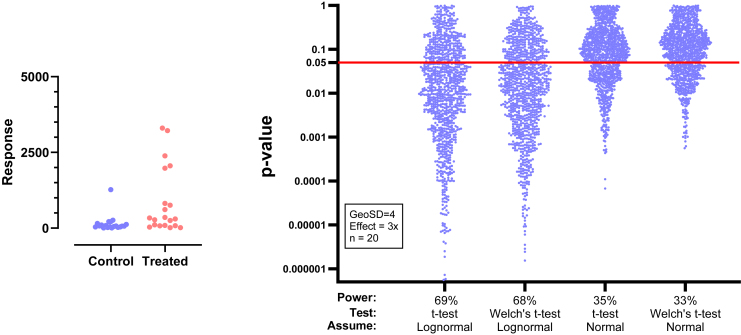
Loss of statistical power when lognormal distributions are analyzed as normal. Left: A simulated experiment with *n* = 20 per group, sampled from lognormal distributions with GeoSD = 4, and the true effect size is 3× (the treated GeoMean is 3 times the control GeoMean). Right: Results from 1000 simulated experiments with *P* values from 4 analyses (from left to right): *t* test assuming lognormal data, Welch’s *t* tests assuming lognormal data, *t* test assuming normal data, and Welch’s *t* test assuming normal data. The powers (the fraction of the *P* values < .05) are shown below each lane. The raw data are in Supplemental Material.

Figure 27 presents additional simulations demonstrating that analyzing lognormal data as normal results in a loss of statistical power. The simulated experiments had *n* = 20 per group. All values were sampled from lognormal distributions with GeoSD = 4, and the true effect size is 3× (the treated GeoMean is 3 times the control GeoMean). The left side of the figure shows 1 of the 1000 simulations. The right side shows *P* values for 1000 simulations.

All 1000 *P* values are graphed for all 4 analyses. For each test, the *P* values vary over several orders of magnitude, more variability than many scientists expect. Statistical power is defined as the probability that the *P* value will be < .05 (or any chosen cutoff), so is the fraction of the dots below the red line that defines *P* = .05. Analyzing the data correctly (assuming lognormal distributions) results in 69% power with the lognormal *t* test and 68% with the lognormal Welch’s *t* test. Analyzing the data incorrectly (assuming normal distributions) yields only 35% with the *t* test and 33% with the Welch’s *t* test. Similar simulations were reported by Fayers (2011).

##### c Increased sample size requirement (for constant power)

Another way to assess how much it matters to identify lognormal variables is to see how it impacts the calculation of necessary sample size.

Let us assume we are sampling from 2 lognormal distributions and running a 2-sample *t* test. The control GeoMean is 100 and we are seeking sample size to detect a doubling to a GeoMean of 200. Assume GeoSD = 3, and use conventional values for *α* (0.05, 2-sided) and desired power (80%).

Because the data are lognormal, a *t* test would be run on the logarithms of the values, so we need to convert to log scale before calculating necessary sample size. On a log scale, the hypothetical means are log_10_(100) = 2 and log_10_(200) = 2.3, and the expected SD = log_10_(3) = 0.477. For these parameters, sample size calculators such as GraphPad Prism Cloud’s Power Analysis calculator or G∗Power (Faul et al, 2007) report the required sample size of 41 per group. Wolfe and Carlin (1999) presented equivalent calculations.

But what if we wrongly assume the data are sampled from normal distributions? This is a contrived situation, but let us do our best to do the corresponding sample size calculations assuming normal distributions. Using the equation shown below, the corresponding AMeans are 224.2 (control) and 448.4 (treated), and the corresponding SDs are 282.8 (control) and 565.7 (treated). The SDs differ because with lognormal data with fixed GeoSD, the SD will be larger when the GeoMean is larger. G∗Power and Prism Cloud’s Power Analysis calculator both allow you to specify different hypothetical SD values for the 2 populations. For this experimental design, both calculate that the resulting necessary sample size is 64 per group.

$$AMean=GeoMean\cdot e^{\frac{{\ln\left(GeoSD\right)}^{2}}{2}}$$

$$SD=\sqrt{{GeoMean}^{2}\cdot e^{{\ln\left(GeoSD\right)}^{2}}-1}$$

**Fig. 28:**
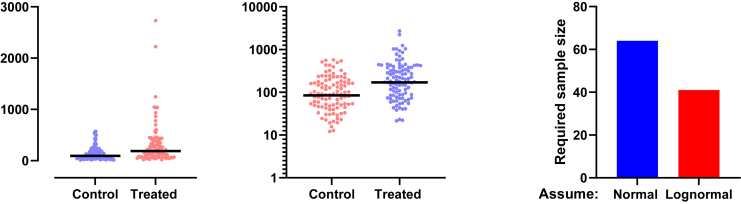
Recognizing lognormality leads to smaller required sample size. The left and middle graphs show 100 values sampled from 2 hypothetical lognormal distributions with GeoSD = 3 with GeoMeans = 100 and 200. The graph on the right shows the required sample sizes (per group) computed correctly assuming the data are lognormal or incorrectly assuming sampling from normal distributions, setting *α* to 0.05 and desired power to 80%. The raw data are in Supplemental Material.

To summarize this contrived example: assuming a normal distribution requires a larger sample size: 64 per group versus 41 per group than if you assume the data are lognormal, a 56% increase. Figure 28 shows samples from the 2 hypothetical populations on linear and logarithmic axes, and the right panel shows the needed sample size (per group) when computed correctly (assuming lognormal) and incorrectly (assuming normal).

Limpert and Stahel (2011) used simulations with a variety of GeoSD values (1.5–3.5) and a variety of intended effect sizes, in order to determine the degree to which sample size can be reduced by recognizing lognormal distributions. They showed that incorrectly assuming that lognormal data were sampled from normal distributions resulted in an increase of necessary sample size from somewhere between 20% and 300% depending on GeoSD and effect size. One of their examples assumed GeoSD = 2.4 (similar to our GEOSD = 3), and they found that mistakenly analyzing the data as if they were sampled from a normal distribution raised the required sample size from 10 to 16, a 60% increase (which matches Fig. 28).

#### 3 Switching to the Welch’s *t* test does not solve the problem

The SDs in Fig. 1 differ considerably between the control and treated groups. This is often the case for lognormal data, even when the *GeoSDs* are identical, because the SD of a lognormal distribution depends on both its GeoMean and GeoSD. These unequal SDs might tempt researchers to replace the usual unpaired *t* test with Welch’s *t* test, as this does not assume the variances (or SDs) of the groups being compared are equal (Rasch et al, 2011; Delacre et al, 2017).

With these sample data the results of the Welch’s *t* test (without log transformation) are nearly identical to those of the regular *t* test. The *P* values (two-tailed) from both tests are .22. The CIs for the difference between means are also quite similar (*t* test, −175 to 739; Welch’s *t* test, −179 to 743).

Because the Welch’s *t* test does not assume the SDs are equal, some might expect it to have more power with lognormal data. In fact, its power is a bit less than the *t* test with lognormal data (Zimmerman and Zumbo, 1993; de Winter, 2016). Another reason to avoid the Welch’s *t* test with lognormal data is that the observed type I error rate of the Welch’s *t* test applied to data sampled from skewed distributions can be much higher than the preset value of *α* (Ahad and Yahaya, 2014).

## VIII Other comparisons of lognormal data

### A Paired *t* test of lognormal data

**Fig. 29:**
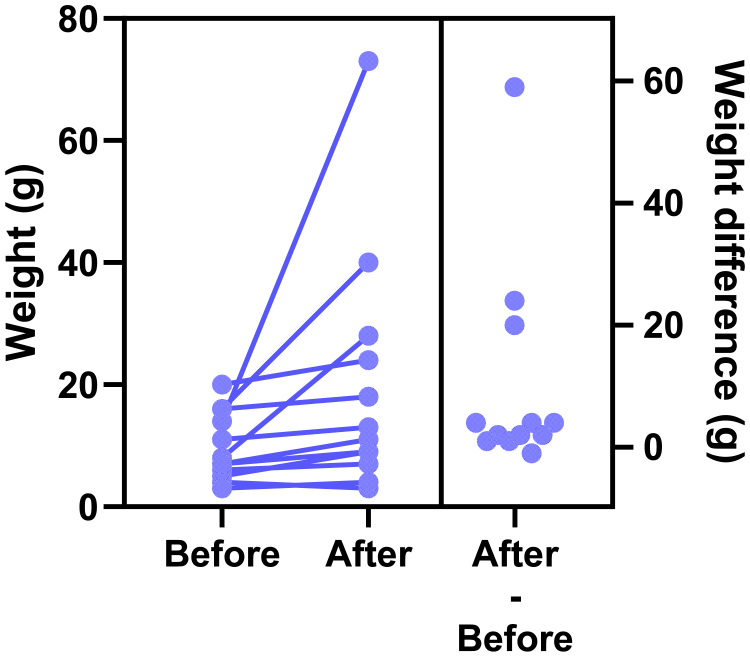
Sample data for paired *t* test. Left: Raw data before and after an intervention; each line is a different animal. The values are in Supplemental Material. Right: The difference (After – Before) for each animal.

Figure 29 shows animal weight before and after an intervention (left panel) and the absolute difference for each animal (right panel). The raw data are in Supplemental Material. Note that the set of differences is skewed, and appears to be lognormal (in fact, the data were simulated so the differences *are* lognormal).

#### 1 Wrong analysis: Paired *t* test of untransformed data

The paired *t* test looks at the set of differences and the null hypothesis that there is no effect of the intervention, in other words that all the variation is due to normal random sampling. The *P* value is .07 (two-tailed). The mean difference is 10.2 g with a 95% CI ranging from −0.80 to 21.1 g. Because the *P* value is > .05, the 95% CI includes 0.0 (no difference). Interpretation of data always depends on the details. These data give a hint of a weight gain, but this large a gain (or an equally large loss, because the *P* value is 2-sided) would occur in 7% of experiments if the null hypothesis (no difference) is true. The CI (also called compatibility interval) is consistent with a small decrease, no change, or a large increase. Note that this analysis assumes the differences are sampled from a normal distribution.

#### 2 Analysis of log-transformed data assuming lognormal distribution of differences

There are 2 equivalent ways of thinking about running a ***lognormal paired* t *test***.

• Transform all the values to logarithms and then run a paired *t* test in order to test the null hypothesis that the means are equal.
• Compute the ratio of before/after for each animal, transform those ratios to logarithms, and run a 1-sample *t* test to test the null hypothesis that the mean of those logarithms is zero (equivalently, the null hypothesis is that the GeoMean of the ratios is 1.0).

**Fig. 30:**
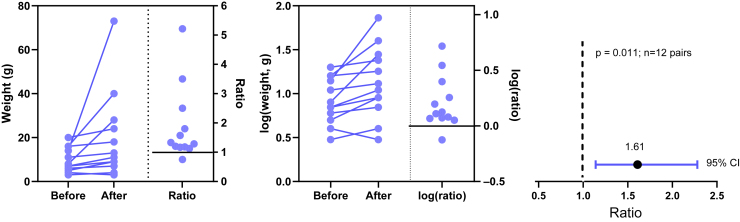
Paired ratio *t* test. Left: Raw data and ratio. Middle: logarithms of raw data and ratio. Right: Summary of ratio *t* test showing: the GeoMean of the ratio, the 95% CI of that ratio, and the *P* value testing the null hypothesis (ie, that the true ratio is 1.0).

These 2 methods are totally equivalent. We prefer the second approach, because it focuses on before/after ratios. Figure 30 shows the set of ratios and log (ratios), and the results of the ratio paired *t* test. We used GraphPad Prism 10.4, but any statistical software would give the same result. The *P* value is .011. The mean log (ratio) is 0.2075, with a 95% CI ranging from 0.0569 to 0.3580. Taking the antilogarithm of all 3 values yields the results as ratios, which are far easier to interpret (see Fig. 30, right panel). The GeoMean of the ratios is 10^0.2075^ = 1.61, with a 95% CI ranging from 1.14 to 2.28. The 95% CI does not include 1.0 (the value that denotes no change), which is consistent with the *P* value being < .05. With GraphPad Prism (version 6.0 and later), choose the *ratio paired* t *test* to obtain the results directly without needing to calculate logarithms and antilogarithms.

Analyzed this way, the data suggest that the intervention changes weight, but this is not super-convincing because the CI is so wide, ranging from a 14% increase to a bit more than a doubling.

### B One-way ANOVA with Dunnett’s test of lognormal data

**Fig. 31:**
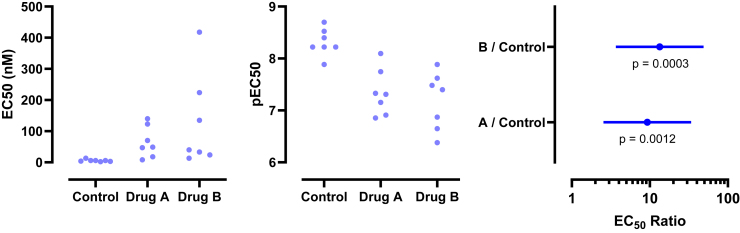
One-way ANOVA example. Left: Raw data as EC50 in nanomolar. The values are in Supplemental Material. Middle: Transformed to pEC50 (convert nanomolar to molar, transform to log10, then multiply by −1). Right: Results of Dunnett’s multiple comparisons test showing the 95% CI for the ratio of EC50s and the *P* values testing the null hypothesis that the true ratio is 1.0. Both CIs and *P* values are corrected for multiple comparisons by the Dunnett calculations.

Figure 31 shows EC50 values collected in control conditions and in the presence of 2 drugs in 7 experiments. In the presence of the drugs, the EC50s tend to be larger so the pEC50s tend to be smaller.

#### 1 Incorrect analysis assuming normal distributions

One-way ANOVA tests the null hypothesis that all 3 data sets are sampled from normal distributions with the same mean and SD. One-way ANOVA of these EC50s results in *P* = .07, high enough that the null hypothesis is not rejected. Welch’s one-way ANOVA, which does not assume equal SDs, results in a slightly higher *P* value (*P* = .11).

#### 2 Analysis assuming lognormal distributions

Here are several reasons to assume these data in Fig. 31 are sampled from lognormal distributions:

• EC50s are generally lognormal (Hancock et al, 1988; Kaumann et al, 1989; Christopoulos, 1998; Walker et al, 2010; Liang et al, 2015).
• The SDs are very different for the 3 data sets (4, 50, and 150), but their CVs are more similar (63%, 77%, and 118%).
• The CVs in all data sets are larger than 60%. As noted earlier, this (plus the fact that negative values are impossible) makes it exceedingly unlikely for the data to be sampled from a normal distribution.

To analyze the data assuming sampling from lognormal distributions, the first step is to transform the values to logarithms. For this example, we converted the data from nanomolar to molar, transformed to log10 logarithms, and then multiplied those logarithms by negative 1 to compute the pEC50. Just as pH is the negative logarithm of [H+], the pEC50 is the negative logarithm of EC50. For example, when the EC50 is 10 nM, which is 10^−8^ molar, the logEC50 is −8, and the pEC50 is 8. pEC50s are often used by pharmacologists to avoid negative numbers.

The middle panel (see Fig. 31) plots the pEC50 in the 3 groups. One-way ANOVA of these values resulted in *P* = .0003 (compare to *P* = .07 from ANOVA on untransformed data). Now focus on the right panel of Fig. 31. The follow-up Dunnett’s multiple-comparison test compares the result of each drug to the control. Dunnett’s test reports the CI for the difference between logarithms. We transformed the confidence limits to their antilogarithm to plot the ratio of EC50s. For control versus drug A, the difference in logarithms is 1.13 with a 95% CI ranging from 0.56 to 1.69. Transform all 3 values to their antilogarithm (10 to those powers) and the ratio of EC50s is 13.5, with the 95% CI ranging from 3.63 to 49.0. Because those CIs do not come close to 1.0 (the value that signifies no effect), the *P* values are much < .05.

For comparing 2 lognormal data sets, our simulations showed the advantage of using the lognormal Welch’s *t* test (see section The lognormal Welch’s t test). We suspect there are similar advantages to using Welch’s ANOVA when analyzing the logarithms of lognormal data, but we have not run any simulations to test this idea.

### C Two-way ANOVA of lognormal data

#### 1 Example and analysis assuming sampling from normal distributions

**Fig. 32:**
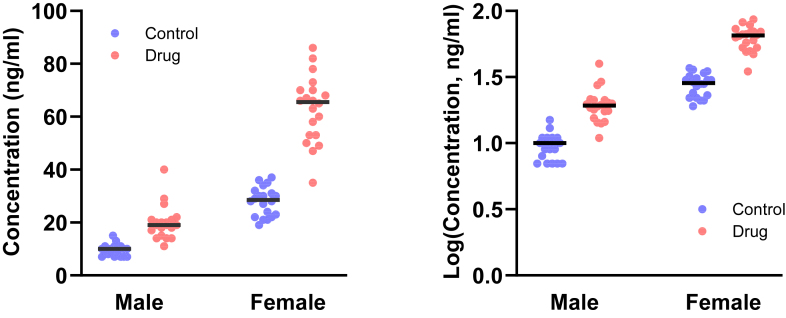
Results of simulated experiment asking whether there is an interaction between drug response and sex. The values are in Supplemental Material. The solid horizontal lines represent the arithmetic means.

Suppose nicotine increases circulating levels of a certain hormone, and you wish to know whether the drug effect is different in males and females. You measure hormone levels in control and drug-treated animals, in both sexes. The effect seems much larger in females (Fig. 32).

To demonstrate and quantify this result, run two-way ANOVA and focus on the results for interaction, which assesses whether the drug effect differs between male and female animals. The interaction *P* value is tiny (<.0001). The drug effect is 25 ng/mL greater in females, with a 95% CI ranging from 18 to 32 ng/mL. This would be convincing evidence of a SEX by DRUG interaction—if all the assumptions behind the analysis are true.

• ANOVA assumes all values are sampled from normal distributions. This is not obviously false, but there is a hint of asymmetry.
• ANOVA assumes that the underlying populations are not only normal but that they all have the same SD. Here, the variability (SD) clearly differs substantially between groups, violating a major assumption underlying ANOVA.

#### 2 Two-way ANOVA assuming sampling from lognormal distributions

But… could the data be lognormal? There are reasons to think so:

• The measurement is concentration, a ratio variable which is often lognormal.
• The variation is larger when the mean is larger, as expected for lognormal data.
• The largest value in the Male/Drug group is quite a bit larger than the others, and is identified as an outlier by Grubbs’ outlier test (*P* < .01). But Grubbs’ test assumes the data (except for the possible outlier) are sampled from normal distributions. Values larger than the others are expected in data sampled from lognormal distributions.

For these reasons, especially the first, it makes sense to assume lognormality, not normality. Can normality and lognormality tests help decide? Not in this case. The data from the drug-treated males fail 3 normality tests but pass all 3 lognormality tests, but the remaining 3 data sets pass 3 normality tests (*P* > .05) and also pass 3 lognormality tests.

The right side of Fig. 32 shows the log-transformed data. Now the variation is similar across conditions, there are no longer any obvious outliers, and the data look normally distributed.

Two-way ANOVA on the log-transformed data shows no evidence of interaction (*P* = .343), so it makes sense to look at the row (sex) and column (drug) effects. Tests of both null hypotheses (that sex makes no difference, and that the treatment makes no difference) result in *P* < .0001. The effect of the drug is shown in the ANOVA results as the difference between the mean of the log-transformed control and drug-treated values. It is reported as 0.3301 (95% CI, 0.2852–0.3751). Use the 10ˆ (ie, 10 to the power of) transform on all 3 values to express the drug effect as a ratio. The drug-treated animals had a response 2.1 times that of the control animals (95% CI, 1.9–2.4). Similar calculations show that the females had a response 3.1 times larger than the males (95% CI, 2.8–3.4).

This example demonstrates:

• How one can be fooled by analyzing lognormal data as if the values were sampled from normal distributions.
• Why it is important to differentiate additive effects from multiplicative effects. Analyzing the raw data (left panel) suggested a substantial 2-way additive interaction, that is, with a larger drug effect in females. However, analysis of the log-transformed data (right panel) revealed no multiplicative interaction.
• That the decision of whether to assume sampling from lognormal distributions cannot depend entirely on normality and lognormality tests.

In the example of Fig. 32, analyzing the data properly (after log transformation) prevented falsely concluding there was an interaction. The converse can also happen: in some cases, analyzing the data after log transformation reveals a multiplicative interaction that would have been missed had the data not been transformed.

## IX Additional topics

### A How to handle values that are zero, negative, or below the limit of detection

#### 1 If some values are zero or negative (occurs rarely)

By definition, an ideal lognormal distribution comprises only positive values. However, in the real world, some data sets that are close to lognormal nevertheless can contain values that are zero or negative. This can occur in 3 ways:

• Zeros can occur when the variable is a count, for example, number of immunopositive cells in a tissue section, or number of days with rainfall. Lognormal distributions describe continuous variables, so really are not appropriate for variables that are counted. The analysis of such data should not be based on assuming sampling from a lognormal distribution.
• Zeros can occur when the variable has a distribution that is not entirely lognormal. One example is the Comet assay, used for detecting DNA damage in eukaryotic cells (Bright et al, 2011). The method uses gel electrophoresis to quantify the fraction of the DNA that has been fragmented so appears in the tail of the “comet.” Combining data from many cells, the distribution is a cluster of zeros plus a collection of values from a lognormal distribution. Special methods are needed to analyze such data.
• Subtracting a baseline or nonspecific value can lead to a difference that is zero or negative. The true population value may be larger than zero, but experimental (ie, random) error in total and/or baseline values can result in a zero or negative difference. It is probably best to analyze such data without subtracting a baseline or nonspecific signal (and fit the baseline in the analysis). Another approach is to add a positive constant to each value in the data set, so that all values become positive before log transformation.

#### 2 If some values are below the detection limit (occurs rarely)

With some experimental systems, a value may be too low to measure. You know the value cannot be zero (or negative), and that it is smaller than the limit of detection (LOD). Such values are called *left-censored*. How can such a value be accounted for?

• Deleting such values would bias the results (leading to an increased GeoMean), because only the smallest value(s) would be removed.
• Replacing a “nondetect” value with the LOD would also bias the results (increase the GeoMean) because the actual (unmeasurable) values are all less than the LOD.
• Replacing nondetects with zero is not possible when assuming lognormal distribution, because analyses of lognormal data first take the logarithm of all the values, and the logarithm of zero is not defined.

Replacing nondetects with some value between zero and LOD is the best solution. Verbośek (2011) used simulations of lognormal data with different total sample size sizes and number of nondetects, and recommends assigning a value of LOD/√2 to all values that are less than the LOD.

The topic of how to deal with values too low to measure has been reviewed by Shoari and Dubé (2018), but these authors do not focus on lognormal data. Zhang et al (2009) recommend analyzing data with too-small-to-measure values using nonparametric methods, and explain how to extend common nonparametric tests to data with values below the detection limit. Zhou and Tu (1999) devised a likelihood method for comparing data sets that are a mixture of values from a lognormal distribution plus zeros.

### B Comparing the arithmetic means of lognormal distributions

Some researchers argue for reporting the AMean instead of the GeoMean in certain contexts. For example, Parkin and Robinson (1992) argue that the AMean provides a more meaningful comparison than the GeoMean or median when comparing variables such as pollutant concentrations across locations, where the important consideration is the total mass of pollutant at a given site.

Surprisingly, working with the AMean of lognormal data requires special methods. This is because the asymmetry of lognormal distributions causes random samples to often underrepresent large values. Thus, it is not appropriate to calculate the AMean by adding up all the values and dividing by the sample size, as this tends to underestimate the true population AMean. The following papers describe appropriate procedures to compute the AMean and its CI (Zhou and Gau, 1997; Wu et al, 2003; Olsson, 2005) and to compare AMeans of different groups (Zhou et al, 1997).

### C The GeoMean as an average of ratios

In this article, we explain the use of the GeoMean as a way to summarize a set of values sampled from a lognormal distribution. But the GeoMean is more widely applicable than that. It is the only consistent way to average ratios (Fleming and Wallace, 1986), which makes it essential in areas such as physics and engineering (Mahajan, 2019), finance (https://www.investopedia.com/articles/investing/071113/breaking-down-geometric-mean.asp), and other domains (https://jlmc.medium.com/understanding-three-simple-statistics-for-data-visualizations-2619dbb3677a; Chargin, 2020). This makes sense only when the ratios are unitless because they are the ratio of the same variable measured in 2 conditions, for example, 2 treatments, 2 time points, or 2 genotypes.

This property makes geometric means essential in pharmacology whenever we need to average ratios, whether analyzing relative potencies or measuring fold-changes in receptor expression. The problem with using the AMean to summarize a set of ratios is that the result depends on which group or treatment is chosen as the baseline. For example, consider 2 experiments: in one, drug A is 3 times more potent than drug B, but in the other, it is only one-third as potent. The AMean of these ratios is (3 + 1/3)/2 = 1.67, implying that drug A is, on average, 1.67 times more potent than drug B. If we instead measure the potency of B relative to A, we arrive at the opposite conclusion—that B is 1.67 times more potent than A. This inconsistency can lead to misleading interpretations.

In contrast, the geometric mean of 3.0 and one-third is 1.0, correctly showing that neither drug is consistently more potent. The GeoMean is a baseline-independent and consistent summary, making it the appropriate method for averaging ratios.

### D Geometric Coefficient of Variation of lognormal data

The CV quantifies variability among values that can only be positive. CV is defined as the SD/AMean. Because SD and AMean are expressed in the same units, the CV is a unitless ratio. It is often multiplied by 100 and reported as a percentage. The smallest possible value of CV is 0.0, which would only occur when all values are identical (no variation).

With lognormal data, variability and asymmetry are intertwined, and the GeoSD quantifies both. A lognormal distribution with more variation is also more asymmetrical. The standard definition of the CV (SD/AMean) can be rewritten as a function of only the GeoSD for data sampled from a lognormal distribution (Koopmans et al, 1964; Ott, 1995; Elassaiss-Schaap and Duisters, 2020).

$$GeoCV=\sqrt{e^{{\ln\left(GeoSD\right)}^{2}}-1}$$

We suggest not reporting the geometric CV (GeoCV) for 2 reasons. First, it adds no information not already expressed by the GeoSD (as GeoCV can be calculated from GeoSD). Second, alternative definitions of GeoCV are in use, leading to inconsistent results. GeoCV has been defined as GeoSD −1 (Kirkwood, 1979), as ln(GeoSD) (Proost, 2019), and as GeoSD/GeoMean (Martinez and Bartholomew, 2017).

### E Geometric standard error of a geometric mean

The GeoSEM of lognormal data is a unitless factor that can be multiplied by or divided into the GeoMean (Kirkwood, 1979). This is defined as the antilogarithm of the SEM of the log (values), which is equivalent to:

$$GeoSEM={GeoSD}^{\frac{1}{\sqrt{n}}}$$

Note the similarity between the definitions of SEM and GeoSEM. SEM equals SD multiplied by (1/√n), whereas the GeoSEM equals GeoSD to the power of (1/√n).

Beware of the earliest definition of the GeoSEM, which had the same units as the data (Norris, 1940). This value was to be added to or subtracted from the GeoMean, which makes little sense for the asymmetrical lognormal distribution.

### F Why skewness is not a useful parameter with lognormal data

**Fig. 33:**
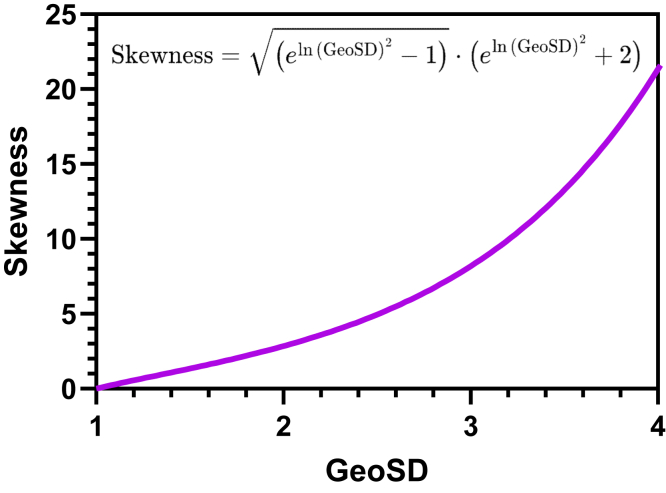
Skewness of a lognormal distribution. The equation in this figure is equivalent to Equation 4.8 in Crow and Shimizu (1988).

Skewness—more precisely *Pearson’s moment coefficient of skewness*, abbreviated *G*_*1*_—quantifies the asymmetry of a distribution. A perfectly symmetrical distribution has a skewness of 0.0. Distributions with a long right tail, including lognormal distributions, have positive skewness. Not surprisingly, there is a simple relationship (Crow and Shimizu, 1988) between the GeoSD and the skewness of a lognormal distribution (Fig. 33).

**Fig. 34:**
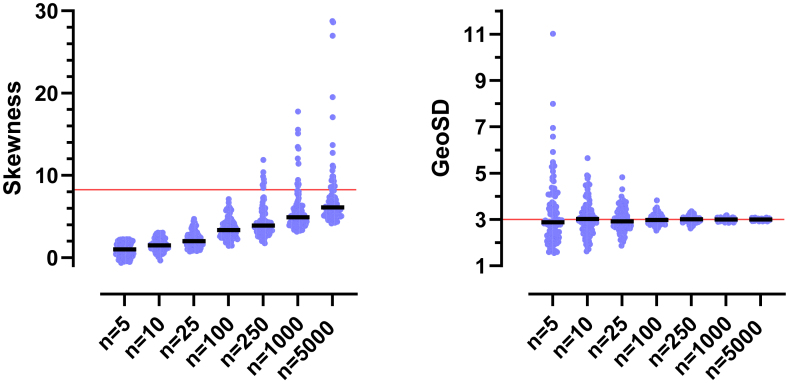
Demonstration that skewness is not a useful measure of asymmetry of a lognormal data set. Each dot represents the analysis of one simulated data set drawn from a lognormal distribution with GeoSD = 3.0 and GeoMean = 10. The left panel shows the values of Skewness from 100 simulated data sets of various sizes, and the right panel shows the values of GeoSD from those same data sets. The horizontal black lines represent the medians. The horizontal red lines mark the population skewness (8.2) and GeoSD (3.0).

Although skewness of a lognormal distribution is related to GeoSD, the left panel of Fig. 34 demonstrates 3 reasons why it is not helpful to quantify the skewness of data sampled from lognormal distributions.

• The figure shows the skewness of 100 simulated data sets sampled from a lognormal distribution with GeoSD = 3.0, which corresponds to skewness = 8.2. But most of the simulated samples have skewness that are much smaller than that (Kirby, 1974; Cox, 2010).
• The maximum possible skewness is limited by sample size (*n*) according to this equation: (*n* − 2)/√(*n* − 1) (Kirby, 1974; Cox, 2010). Therefore, the skewness can be 8.2 or greater only when *n* is 71 or larger. But even huge samples rarely obtain skewness that large. With *n* = 5000 (rightmost column), only 18% of the 100 simulated samples have skewness > 8.2.
• Even with large samples, the skewness varies considerably from sample to sample. Skewness is therefore not a reliable way to assess the asymmetry of data sampled from a lognormal distribution.

In contrast, the right side of Fig. 34 shows that the GeoSD of simulated samples is well behaved. The values are centered on the true GeoSD, and the variation decreases with larger samples.

### G How much is lost when normal data are analyzed as if lognormal?

Analyzing lognormal data as if they were sampled from normal distributions can lead to major problems in data analysis. How bad is the reverse issue: analyzing normal data as if they were sampled from lognormal distributions?

This is a bit tricky to think through because all normal distributions include negative values, which are impossible in lognormal distributions. But if the CV is small enough, only a tiny fraction of samples will contain negative values. Therefore, it is only possible to be unsure about whether data are sampled from normal versus lognormal distributions when the CV is small. In this case, the lognormal distribution looks almost identical to a normal distribution, so the loss of power tends to be minimal (simulations not shown).

### H Performing lognormal comparisons with GraphPad Prism

Although data from lognormal distributions can be analyzed by programming languages such as R and Python, GraphPad Prism is the only statistics program we know of that can directly perform *t* tests and ANOVA with lognormal data. Performing a paired *t* test with lognormal data has been available since version 6. Choose the “ratio paired *t* test.” One- and 2-sample (unpaired) *t* tests and one-way ANOVA can be done with lognormal data starting with version 10.5. Simply select the option to assume sampling from lognormal distributions, and then choose between the lognormal *t* test and the lognormal Welch’s *t* test.

## X Summary

### A Properties of lognormal distributions

• Lognormal distributions arise naturally from multiplicative biological and physical processes, whereas normal distributions come from additive processes. Thus, lognormality often reflects fundamental biological processes and is not just a statistical “trick.”
• All values in a lognormal distribution are positive. Zero and negative values cannot be part of a lognormal distribution.
• Lognormal variables are ratio scale variables. Zero must represent the absence of a quantity (eg, weight or concentration) or an asymptotic limit that can be approached but never reached (eg, EC50 or K_m_).
• The geometric mean (GeoMean) of an ideal lognormal distribution equals the median and is always smaller than the AMean. The GeoMean of data randomly sampled from a lognormal distribution provides the best estimate of the population median.
• The GeoSD is a unitless factor that quantifies both spread and asymmetry, and is always > 1.0.
• When the GeoSD is small (less than about 1.3), a lognormal distribution becomes nearly indistinguishable from a normal distribution. Lognormal distributions with larger GeoSDs are skewed, but the skewness may not be obvious with small sample sizes.
• The product or ratio of 2 lognormal variables is also lognormal. This partially explains why so many pharmacological parameters are lognormal.
• When assessing lognormal data, treatment effects are best expressed as ratios rather than differences. This is because a doubling, say, represents the same effect regardless of baseline.

### B Lognormality in pharmacology

• Measurements such as concentration, weight, and enzyme activity are often lognormal.
• Key pharmacological parameters—including EC50, IC50, Kd, K_m_, K_on_, K_off_, clearance, and half-life—follow lognormal distributions.
• The ubiquity of lognormal distributions in pharmacology stems from the multiplicative nature of many chemical and biological processes and also from the fact that a parameter formed as a ratio of 2 lognormal parameters will itself be lognormal.

### C Recognizing lognormal data

• The decision to treat data as lognormal should be based primarily on the nature of the variable and prior experience, and not on the result of normality or lognormality tests.
• For a variable that can only be positive: when the CV is greater than about 0.6, the data cannot be sampled from a normal distribution because more than 5% of the values would be negative.
• With lognormal data, if the GeoSD remains consistent for all groups, expect the SD to vary proportionally with the group mean.
• Many data sets, especially with small sample sizes and small GeoSD, pass both normality and lognormality tests, making these tests often unreliable for distribution decisions.

### D Analyzing lognormal data

• Correctly recognizing lognormality can result in a smaller required sample size (with the same power). The statistical power advantage of recognizing lognormality increases with larger GeoSD values—when GeoSD exceeds 2.0, analyzing data as if normal can reduce power by 50% or more.
• Outlier tests that assume a normal distribution are frequently misleading when applied to untransformed lognormal data.
• Using the Welch’s *t* test on the log-transformed values is recommended. Using the Welch’s *t* test with untransformed lognormal data can lead to misleading results, as this approach can result in an elevated type I error rate and decreased power.
• If a nonparametric approach is required, use the Brunner-Munzel test, which handles asymmetrical distributions better than the Mann-Whitney test.
• Do not rely on AMean ± SD error bars for lognormal data, as they can be misleading when the true variation is asymmetrical. In some cases, the lower error bar can even descend to an impossible negative value.
• Express experimental effects on lognormal variables as ratios. A 75% decrease in EC50 represents the same effect size regardless of the baseline EC50, just as a doubling in enzyme activity represents the same effect size regardless of the baseline activity. Avoid the ambiguous terms *fold change* and *percentage change.*

### E Common misconceptions

**Misconception**: Lognormal distributions are rare special cases.

**Reality**: They are common.

**Misconception**: Data should be considered normal until proven lognormal.

**Reality**: For variables that must be positive, lognormal distribution is often more likely.

**Misconception:** Lognormal distributions are always obviously skewed.

**Reality**: With small GeoSD, they can be nearly symmetrical and look very similar to normal distributions.

**Misconception**: Effects should always be presented as absolute differences.

**Reality**: For lognormal variables, ratios are more meaningful because they represent the same effect regardless of baseline.

**Misconception**: Log transformation is a form of p-hacking (invalid data manipulation).

**Reality**: It is a valid statistical choice when justified by the nature of the variable and should be prespecified in analysis plans.

**Misconception:** If a data set passes a normality test, the data cannot be sampled from a lognormal distribution.

**Reality:** Many data sets pass both normality and lognormality tests (especially with small sample sizes).

**Misconception**: Standard outlier tests work for any distribution.

**Reality**: These tests are invalid for untransformed lognormal data and can lead to inappropriate exclusion of legitimate high values.

**Misconception**: Reporting lognormal analyses requires that your readers are facile with logarithms.

**Reality**: Results can be presented in original units using ratios and GeoMeans without mentioning logarithms.

**Misconception**: CIs are always symmetrical.

**Reality**: For lognormal data, the CIs of a GeoMean and the CI of a ratio of 2 GeoMeans are asymmetrical.

### F Perspective

Lognormal distributions are not merely a statistical curiosity—they are fundamental to how biological and pharmacological processes behave. When multiple factors influence a biological outcome through multiplication rather than addition, lognormal distributions naturally emerge.

Although the mathematical foundations may appear daunting, the core concepts are intuitive, and the practical implications are profound. Recognizing and properly accounting for lognormal distributions results in smaller sample sizes, more reliable outlier detection, and more meaningful presentation of results. Most important, assuming lognormal distributions shifts thinking about experimental effects from differences to ratios, and this often aligns better with biological reality—a doubling of enzyme activity (or a halving of drug potency, say) represents the same effect regardless of baseline values.

To emphasize these points playfully, we conclude with a poem in the style of Dr Seuss.

**Oh the lognormal insights you will gain!**

When *your* data’s askew,

And you don’t know what to do.

When your values spread wide,

All on the positive side.

For binding and clearance, EC50s galore,

For enzyme kinetics and so much more,

They multiply, multiply, that’s nature’s way!

Not adding like normal statistics would say.

By welcoming lognormal, you’re thinking grows clear,

Required sample size shrinks, no false outliers here.

Simple ratios illuminate the way,

While absolute differences lead our insights astray.

When processes multiply rather than add,

Normal statistics can make results look bad.

Log-transform your data so analyses can thrive,

Oh the insights you’ll gain, and the wisdom you’ll derive!

## Declaration of generative AI and AI-assisted technologies in the writing process

During the preparation of this work the author(s) used Claude.ai 3 to enhance the manuscript’s clarity and conciseness, verify citation-reference consistency, and generate alternative versions of the concluding poem. After using this tool, the author(s) reviewed and edited the content as needed and take(s) full responsibility for the content of the publication.

## Conflict of interest

Harvey J. Motulsky is the founder of GraphPad Software (creator of Prism) and a minority shareholder of the company that now owns it. Trajen Head is the Senior Product Manager for Prism, and is a minority shareholder of the company that owns GraphPad Software. Paul B.S. Clarke declares no conflict of interest.
