# Supplementary material for: Analyzing lognormal data: A nonmathematical practical guide
Source: Pharmacol Rev. 2025 Feb 25;77(3):100049. doi: 10.1016/j.pharmr.2025.100049 (PMC12163497; doi:10.1016/j.pharmr.2025.100049)
Supplement: Supplementary Material [file mmc2.docx]

A supplement to: Motulsky and Clarke, Analyzing Lognormal Data: A Nonmathematical Practical Guide, Pharmacological Reviews

# Derivation of the equation to calculate the likelihood ratio of data being sampled from lognormal vs. normal distribution from the SD, GeoSD, AMean, and GeoMean

### Defining the likelihood ratio

Burnham and Anderson (2002) presented an equation to compute the relative likelihood of a data set being sampled from a normal or lognormal distribution.

The likelihood that data would be sampled from a normal distribution is:

$$\mathcal{L}_{normal}= \left[ \frac{1}{\sqrt{2\pi}} \right]^{n}\left[ \frac{1}{s_{y}} \right]^{n}exp\left[ \frac{{\sum_{i=1}^{n} \left( y_{i}-m_{y} \right)}^{2}}{-2s_{y}^{2}} \right]$$

In this equation, m_y_ and s_y_ are the mean and sample standard deviation of all n values, and each value is denoted by y_i._

The likelihood that the data would be sampled from a lognormal distribution is:

$$\mathcal{L}_{lognormal}= \left[ \prod_{i=1}^{n} \frac{1}{y_{i}} \right]\left[ \frac{1}{\sqrt{2\pi}} \right]^{n}\left[ \frac{1}{s_{\mathrm{lny}}} \right]^{n}exp\left[ \frac{{\sum_{i=1}^{n} \left( \ln\left( y_{i} \right)-m_{lny} \right)}^{2}}{-2s_{lny}^{2}} \right]$$

In this equation, m_lny_ and s_lny_ are the mean and sample standard deviation of the natural logarithm of all n values, and each value (original, not log transformed) is denoted by y_i._

The ratio of the two likelihoods (LR) is:

$$LR=\frac{L_{normal}}{L_{lognormal}}=\frac{\left[ \frac{1}{\sqrt{2\pi}} \right]^{n}\left[ \frac{1}{s_{y}} \right]^{n}exp\left[ \frac{{\sum_{i=1}^{n} \left( y_{i}-m_{y} \right)}^{2}}{-2s_{y}^{2}} \right]}{\left[ \prod_{i=1}^{n} \frac{1}{y_{i}} \right]\left[ \frac{1}{\sqrt{2\pi}} \right]^{n}\left[ \frac{1}{s_{\mathrm{lny}}} \right]^{n}exp\left[ \frac{{\sum_{i=1}^{n} \left( \ln\left( y_{i} \right)-m_{lny} \right)}^{2}}{-2s_{lny}^{2}} \right]}$$

Since the likelihood of a normal distribution is in the numerator, the LR will be greater than 1.0 when the data are more likely sampled from a normal than a lognormal distribution, and will be less than 1.0 when the lognormal distribution is more likely.

### Simplifying the equation

Burnham and Anderson (2002) stop here, but the equation can be simplified. Here we simplify it greatly into a simple combination of the arithmetic and geometric means, and the arithmetic and geometric standard deviations.

The definitions of the variances are:

$$s_{y}^{2}=\sum_{i=1}^{n} \frac{\left( y_{i}-m_{y} \right)^{2}}{n-1}$$

$$s_{lny}^{2}=\sum_{i=1}^{n} \frac{\left( ln\left( y_{i} \right)-m_{lny} \right)^{2}}{n-1}$$

Substituting:

$$LR=\frac{L_{normal}}{L_{lognormal}}=\frac{\left[ \frac{1}{\sqrt{2\pi}} \right]^{n}\left[ \frac{1}{s_{y}} \right]^{n}exp\left[ -\frac{1}{2}\left( n-1 \right) \right]}{\left[ \prod_{i=1}^{n} \frac{1}{y_{i}} \right]\left[ \frac{1}{\sqrt{2\pi}} \right]^{n}\left[ \frac{1}{s_{\mathrm{lny}}} \right]^{n}exp\left[ -\frac{1}{2}\left( n-1 \right) \right]}$$

Cancelling the identical pi and exp terms:

$$LR=\frac{L_{normal}}{L_{lognormal}}=\frac{\left[ \frac{1}{s_{y}} \right]^{n}}{\left[ \prod_{i=1}^{n} \frac{1}{y_{i}} \right]\left[ \frac{1}{s_{\mathrm{lny}}} \right]^{n}}=\frac{\left[ \frac{1}{s_{y}} \right]^{n}}{\left[ \frac{1}{\prod_{i=1}^{n} y_{i}} \right]\left[ \frac{1}{s_{\mathrm{lny}}} \right]^{n}}$$

Simplify further by substituting the definition of the GeoMean and GeoSD.

$$GeoMean=\left[ \prod_{i=1}^{n} y_{i} \right]^{\frac{1}{n}} so \prod_{i=1}^{n} y_{i}={GeoMean}^{n}$$

$$GeoSD=e^{s_{lny}}\mathrm{so}s_{lny}=\ln\left( GeoSD \right)$$

$$LR=\frac{L_{normal}}{L_{lognormal}}=\frac{\left[ \frac{1}{s_{y}} \right]^{n}}{\frac{1}{{GeoMean}^{n}}\left[ \frac{1}{\ln\left( GeoSD \right)} \right]^{n}}=\frac{{{GeoMean}^{n}\cdot\ln\left( GeoSD \right)}^{n}}{{s_{y}}^{n}}$$

$$=\left[ \frac{GeoMean\cdot\ln\left( GeoSD \right)}{s_{y}} \right]^{n}=\left[ \frac{\mathrm{GeoMean}}{s_{y}}\cdot\ln\left( GeoSD \right) \right]^{n}$$

The definition of the coefficient of variation is

CV = S_y_/Arithmetic Mean, so S_y_ = CV*Arithmetic Mean

Therefore:

$$LR=\left[ \frac{GeoMean\cdot\ln\left( GeoSD \right)}{CV*Arithmetic Mean} \right]^{n}=\left[ \frac{GeoMean}{Arithmetic Mean}\cdot\frac{\ln\left( GeoSD \right)}{CV} \right]^{n}$$

This equation makes sense:

- The GeoMean and arithmetic mean are both measures of central tendency expressed in the same units, so their ratio is unitless. The CV and ln(GeoSD) are both unitless measures of variation, so their ratio is also unitless. Therefore LR has no units.
- Sample size (n) has the expected impact. The product of the mean ratio (GeoMean/AMean) and the variability ratio [ln(GeoSD)/CV] is taken to the power of n. A larger sample size will push the likelihood ratio (LR) further away from 1.0, reflecting the increased evidence provided by more data points.
- When data are sampled from a lognormal distribution, the arithmetic mean is pulled higher than the geometric mean because of the long right tail, and the CV is usually larger than ln(GeoSD). Both ratios are therefore usually less than 1.0, as is their product.
- When data are sampled from a normal distribution, the GeoMean is usually slightly smaller than the AMean (due to the symmetrical distribution), and the CV is usually smaller than ln(GeoSD). Both ratios are therefore usually greater than 1.0, as is their product.
- The LR only compares the relative fit of a normal vs. lognormal distribution. If the true underlying distribution is neither normal nor lognormal, the LR will still favor one of these two options.

### Probability the data came from normal vs. lognormal distribution

What are the probabilities the data came from a normal vs. a lognormal distribution? If you assume equal prior probabilities of sampling from a normal vs. a lognormal distribution and ignore the possibility that the data might come from a different distribution, the probability the data were sampled from a normal distribution is:

$$P_{Normal}=\frac{L_{normal}}{L_{normal}+L_{lognormal}}=\frac{LR}{\left( 1+LR \right)}$$

The probability that the data were sampled from a lognormal distribution is therefore:

$$P_{lognormal}=1-P_{Normal}$$

## Reference

Burnham K, and Anderson D (2002) *Model selection and multimodel inference: a practical information-theoretic approach*, Second edition, Springer.
