# Supplementary material for: Analyzing lognormal data: A nonmathematical practical guide
Source: Pharmacol Rev. 2025 Feb 25;77(3):100049. doi: 10.1016/j.pharmr.2025.100049 (PMC12163497; doi:10.1016/j.pharmr.2025.100049)
Supplement: Supplementary Material [file mmc4.docx]

A supplement to: Motulsky and Clarke, Analyzing Lognormal Data: A Nonmathematical Practical Guide, Pharmacological Reviews

## Simulations demonstrate that the distributions of EC50, K_m_, B_max_, Hill slope, and rate constants are closer to lognormal than normal

Method

All the graphs in this Supplement were created using the following steps with GraphPad Prism 10.3. Simulate an experiment with ideal data and no experimental error. Add random error to each value from a normal distribution with mean=0 and standard deviation (SD) equal to 10% or 20% of the Y value (as specified in figure legend). Fit the model to the data with weighted nonlinear regression to minimize the sum of squares of the residual divided by Y. All the parameters were fit; none were fixed to constant values. Repeat 10,000 times, graph the frequency distribution of the parameter values (green bars), fit both normal (dotted red) and lognormal (solid blue) distributions, and tabulate both R^2^ values and the difference between the two AICc (Akaike Information Criterion corrected for small sample sizes) values.

## Concentration-response curves

Y=Bottom + (Top-Bottom)/(1+10^((LogEC50-X)*HillSlope))


**Figure 1.** Left: A simulated concentration-response curve with random scatter sampled from a normal distribution with SD=10% of Y. Right: Both EC50 and slope factor are fit better by the lognormal (red dotted) than the normal (blue solid) distribution.

## Enzyme kinetics and equilibrium binding

Y=Vmax*X/(Km+X)

**Figure 2.** Left: A simulated enzyme velocity curve with random scatter sampled from a normal distribution with SD=20% of Y. Right: Both K_m_ and V_max_ are better by the lognormal (blue solid) than the normal (red dotted) distribution (but for V_max_, the two are very similar).

## Dissociation kinetics

Y=Ymax*exp(-Koff*X)

**Figure 3.** Left: A simulated dissociation kinetics curve with random scatter sampled from a normal distribution with SD=20% of Y. Right: K_off_ is fit better by the lognormal (blue solid) than the normal (red dotted) distribution.

## Association kinetics

Y=Y_0_ + (Plateau-Y_0_)*(1-exp(-Kon*X))

**Figure 4.** Left: A simulated association kinetics curve with random scatter sampled from a normal distribution with SD=20% of Y. Right: K_on_ is fit better by the lognormal (blue solid) than the normal (red dotted) distribution.

## Quantifying the superiority of the lognormal distribution for all parameters we assessed

This table compares the fits of the normal and lognormal distributions to the distributions of the parameters.

|  | **R^2^** | | **ΔAICc** |
| --- | --- | --- | --- |
|  | **Normal** | **Lognormal** | **N-LN** |
| **EC50** | 0.9836 | 0.9984 | 185 |
| **Slope Factor** | 0.9903 | 0.9982 | 80 |
| **K_m_** | 0.9730 | 0.9926 | 49 |
| **V_max_** | 0.9941 | 0.9971 | 30 |
| **k_off_** | 0.9861 | 0.9986 | 1163 |
| **k_on_** | 0.9918 | 0.9965 | 41 |

In all cases, R² is higher for the lognormal distribution.

The support of data for two alternative models is quantified with the difference in AICc, calculated as AICc of the normal distribution minus the AICc of the normal distribution. In all cases, that difference is positive, demonstrating that the lognormal distribution is the preferred model. A difference of greater than 10 would mean that the worse-fitting model (normal distribution) has essentially no support from the data (Burnham and Anderson, 2002; Portet, 2020). Here the smallest ΔAICc is 30, demonstrating the lognormal distribution fits substantially better for all the parameters.

The concentrations of Fig. 1 were equally spaced on a logarithmic axis, while the concentrations in Fig. 2 were equally spaced on a linear axis. The distributions of EC_50_ (Fig. 1) and K_m_ (Fig. 2) are both lognormal (at least approximately). This demonstrates that the lognormality is based on the nature of the model being fit, and not on the spacing of the concentrations in the experimental design.

## The uncertainty of an EC50 from a single experiment is approximately lognormal

Figure 5 shows a sigmoidal (logistic) curve fit to the simulated data with nonlinear regression to find the top and bottom plateaus, the slope factor, and the EC_50_. These values were simulated with few data points and lots of scatter, so the confidence interval would be wide. The confidence intervals were computed using the *profile likelihood* method (Venzon and Moolgavkar, 1988; Royston, 2007; Lee and Bae, 2023) using GraphPad Prism. This method determines the confidence interval of a parameter without making any assumption about the underlying probability distribution. The profile likelihood confidence interval of the EC_50_ is symmetrical on a logarithmic axis (left panel), and so is also nearly symmetrical on a multiplicative scale. The upper confidence limit is 2.2 times the EC50, and the lower confidence limit equals the EC50/2.2. But it is quite asymmetrical on a linear (concentration) scale (right panel). This demonstrates that the sampling error of the EC_50_ is much closer to lognormal than normal.

**Figure 5. The EC_50_ from a single experiment has lognormal uncertainty.** Left: Concentration-response curve with a green band showing the profile likelihood 95% CI for the EC_50_ on a logarithmic axis. Right: The 95%CI of the EC_50_ shown on a linear scale where it is quite asymmetrical. The EC50 is 1.3 µM, and the 95% CI extends from 0.58 µM to 2.6 µM .

## References

Burnham K, and Anderson D (2002) *Model selection and multimodel inference: a practical information-theoretic approach*, Second edition, Springer.

Lee MH, and Bae K-S (2023) Likelihood interval for nonlinear regression. *Transl Clin Pharmacol* **31**:85–94.

Portet S (2020) A primer on model selection using the Akaike Information Criterion. *Infect Dis Model* **5**:111–128.

Royston P (2007) Profile likelihood for estimation and confidence intervals. *Stata Journal* **7**:376–387.

Venzon DJ, and Moolgavkar SH (1988) A method for computing profile-likelihood-based confidence intervals. *Applied Statistics* **37**:87.
